# Assessing the Value for Money of AI-Assisted Technologies for Older Adults: Scoping Review of Economic Evaluations

**DOI:** 10.2196/104019

**Published:** 2026-09-30

**Authors:** Qi Gao, Minji Hong, Yot Teerawattananon, Yi Wang

**Affiliations:** 1 Saw Swee Hock School of Public Health, National University of Singapore and National University Health System, 12 Science Drive 2, Singapore, 117549, Singapore, 65 6516 4988; 2 Health Intervention and Technology Assessment Program, Nonthaburi, Thailand

**Keywords:** digital health, artificial intelligence, older health care, scoping review, cost-effectiveness analysis

## Abstract

**Background:**

As populations age globally, AI-enabled digital health interventions (DHIs) are increasingly being adopted to support integrated, person-centered care for older adults. However, despite rapid advances in AI technologies, their economic value in older care remains poorly understood.

**Objective:**

This scoping review aimed to map the existing literature on the economic evaluations of AI technologies for older adults by (1) identifying the types, characteristics, economic outcomes, and methodological approaches reported; (2) mapping the evidence across the World Health Organization’s (WHO) Integrated Care for Older People (ICOPE) pathway; and (3) identifying evidence gaps and priorities for future research.

**Methods:**

A scoping review was conducted following the PRISMA-ScR (Preferred Reporting Items for Systematic Reviews and Meta-Analyses extension for Scoping Reviews) guidelines. PubMed, Scopus, Embase, Web of Science, and EconLit were searched for studies published up to July 31, 2026. Eligible studies were economic evaluations of AI-based technologies in older health care. Conference abstracts, reviews, technical reports, protocols, letters, trial registrations, and non-English studies were excluded. Methodological quality and reporting quality were assessed using the Criteria for Health Economic Quality Evaluation (CHEQUE). Data were synthesized using descriptive statistics and narrative synthesis, with findings mapped to the 4-step ICOPE care pathway.

**Results:**

Forty studies published between 2018 and 2026 met the inclusion criteria. Methodological and reporting quality were generally high (mean scores: 85.7/100 and 85.4/100, respectively), although equity considerations, subgroup heterogeneity, and model validation were frequently underreported. Most evaluations examined AI for screening and diagnosis (32/40), particularly in cancer and ophthalmology, and primarily used model-based approaches, including decision trees, Markov models, and discrete-event simulations. AI-related costs were frequently obtained from assumptions, manufacturer quotes, or expert opinion. Twenty-one of the forty evaluations reported AI interventions to be cost-saving, and another 17 reported AI interventions to be cost-effective. Confidence in these findings is constrained by methodological limitations, reliance on modeled assumptions, and incomplete reporting of AI-related costs. Key drivers of cost-effectiveness included AI performance, AI-related costs, population characteristics, disease burden, and health system context. Along the ICOPE pathway, evidence was concentrated in screening and diagnostic interventions, with little economic evaluation of personalized care planning or long-term monitoring.

**Conclusions:**

This review summarizes the currently available economic evidence on AI-assisted technologies in older health care following the ICOPE care pathway. It extends beyond prior reviews that have assessed AI cost-effectiveness across general or disease-specific populations without addressing the distinct cost structures, care needs, and equity considerations of older adults. By mapping the included evidence against the ICOPE domains, this review identifies where economic evidence is concentrated and where it is critically lacking, providing structured guidance for policy design and future research across different components of the care pathway.

## Introduction

### Rationale

As population aging accelerates worldwide, health care systems face growing demand for chronic disease management, multimorbidity care, and long-term support for older adults. Several frameworks have emerged globally to guide care delivery for the older populations, including the Senior Friendly Care Framework [1], which promotes system-level adaptations across hospital and community settings; the United States-developed clinical decision-making framework centered on 4 domains—What matters, medication, mentation, and mobility [2]; and Singapore’s Geriatric Nursing Competency Framework [3], which defines the knowledge, skills, and attitudes required of nurses in geriatric settings. Apart from these, the World Health Organization’s (WHO) Integrated Care for Older People (ICOPE) framework offers a person-centered pathway integrating screening, assessment, and management of intrinsic capacity across the care continuum to promote healthy aging [4]. However, translating these frameworks into routine practice demands scalable, resource-efficient tools, which highlights the need for innovative solutions to support aging populations across diverse care settings.

Digital health interventions (DHIs), particularly those enabled by AI, have emerged as promising tools to operationalize and support such integrated care models. AI refers to computational technologies that simulate human intelligence to perform tasks such as pattern recognition, learning, and decision-making, encompassing methods including machine learning, computer vision, natural language processing, and automated decision-support algorithms [5]. The rapid expansion of AI in health care offers potential to enhance diagnostic accuracy, streamline clinical workflows, improve care coordination, and support timely, data-driven decisions. Within older care, AI-enabled systems, such as predictive algorithms [6], remote monitoring devices [7], and automated triage tools [8], may reduce provider workload while enabling proactive and continuous care delivery.

Prior reviews have identified multiple functional roles of AI in older care, including rehabilitation support, emotional and social engagement, supervision of daily activities, and cognitive health promotion [9,10]. However, despite the rapid growth of AI applications and the extensive reporting of algorithmic performance, significant gaps remain in the evidence base for applying AI in older care. Existing studies primarily emphasize technical metrics such as diagnostic accuracy, model validity, and computational performance [11-13]. In contrast, much less is known about the economic implications and cost-effectiveness of AI technologies when deployed in real-world older care settings. Understanding these economic dimensions is essential, as interventions that are clinically effective may not necessarily be economically viable or sustainable when deployed at scale. A separate body of reviews has addressed economic evaluations of AI in health care more broadly [14-18]. However, these reviews largely synthesize evidence across heterogeneous populations, diseases, and health care settings, providing limited insight into the unique challenges of older care. Moreover, existing reviews rarely examine how economic evidence is distributed across the continuum of older care [4], making it difficult to determine which stages of care have been adequately evaluated and where important evidence gaps remain.

In this context, Health Technology Assessment (HTA) provides a structured approach to evaluate not only the clinical effectiveness but also the economic, organizational, and equity implications of health technologies [19]. Although a growing number of formal HTA and economic evaluations have been conducted on AI-assisted interventions, the current evidence remains fragmented across settings, populations, and methodological approaches [15]. To date, reviews that comprehensively synthesize and critically appraise this emerging evidence base within older care contexts remain limited.

### Objectives

To address these gaps, this review distinguishes itself from previous work by focusing specifically on the economic evidence for AI-enabled interventions in older care and by organizing that evidence using the ICOPE framework. Rather than simply cataloging economic evaluations, the review maps evidence across the continuum of older adult care from screening and assessment to treatment, rehabilitation, long-term management, and supportive care. By integrating HTA perspectives with the ICOPE framework, this review provides a policy-relevant understanding of whether current economic evidence adequately supports the adoption of AI throughout the older care pathway.

ICOPE was selected as the organizing structure because it encompasses the full spectrum of health and care services, spanning clinical care, community services, and social support, and covers the entire trajectory of older adult care from early screening to long-term management. As a person-centered framework designed to support integrated care across the aging trajectory, ICOPE also provides a meaningful basis for examining whether economic evaluations are aligned with the different stages at which AI technologies are intended to generate value and inform resource allocation. This makes ICOPE a comprehensive and comparative framework that can provide a coherent scaffold for comparing AI applications across distinct stages of older adult care, reflecting the full complexity of aging rather than isolated clinical problems. Mapping economic evaluations to ICOPE further enables a systematic assessment of where evidence is concentrated or lacking along the care continuum, thereby identifying under-evaluated stages that may warrant greater research and policy attention.

Through this mapping, we aim to address the following questions: (1) What is the current evidence base regarding the economic evaluation of AI technologies for older adults, and what methodological and empirical gaps remain? (2) How do AI-assisted interventions perform in terms of cost, cost-effectiveness, and overall economic impact across different clinical applications in older health care? (3) What intervention and contextual factors influence the cost-effectiveness of AI interventions in older health care? The review systematically synthesizes the different challenges of conducting health economic evaluations across distinct types of AI applications. By clarifying where evidence is strong, where it is lacking, and what gaps require further investigation, this study aims to offer practical guidance for future evaluators and inform evidence-based policy and decision-making regarding the sustainable adoption of AI for aging populations.

## Methods

### Protocol and Registration

The review followed the Joanna Briggs Institute (JBI) methodology for scoping reviews [20], with methods developed prior to the literature search and reported in accordance with the PRISMA-ScR (Preferred Reporting Items for Systematic Reviews and Meta-Analyses extension for Scoping Reviews) checklist [21]. The search protocol was adapted from the review methodologies reported by El Arab and Al Moosa [15] and Imran and Khan [22], and was subsequently reviewed with guidance from Ms Annelissa Chin Mien Chew, a research librarian at the Medicine+Science Library, National University of Singapore. This review was not registered in a public registry.

### Eligibility Criteria

The eligibility criteria of this review were defined and summarized in Table 1. AI was defined as a computer-based system capable of performing tasks that typically require human intelligence, such as learning from data, recognizing patterns, making predictions, or supporting decision-making through techniques including machine learning, deep learning, natural language processing, or computer vision. We defined “older adults” as individuals aged ≥50 years to capture early functional decline and increasing multimorbidity risks that are particularly relevant for preventive and AI-enabled interventions. This approach is consistent with global health frameworks [23] that emphasize early decline in intrinsic capacity prior to the conventional threshold of 65 years, recognizing that biological aging varies across demographic groups and across global contexts [24]. As the objective of this review was to evaluate AI technologies for age-related health care, studies enrolling broader adult populations were also eligible if they evaluated interventions intended for aging-related conditions or care pathways and reported results relevant to older adults. For studies reporting age group-specific analyses, only data pertaining to older adults were extracted and synthesized in this review.

**Table 1.** Inclusion and exclusion criteria.

	Inclusion criteria	Exclusion criteria
Population	Adults aged 50 years and older	None
Concept	Studies evaluating AI-assisted or AI-enabled technologies implemented for health care purposes, reporting health economic endpoints such as cost-effectiveness, cost-utility, cost-benefit, cost-minimization, or cost-consequence outcomes.	Studies evaluating technologies not implemented for health care purposes; studies not reporting a health economic endpoint.
Context	Studies conducted in any health care setting, including hospital, community, or home-based care, in any country or region.	Studies in which AI was used solely as an evaluation tool in the economic evaluation process, rather than as the primary intervention under evaluation.
Types of evidence sources	Peer-reviewed publications, evaluation studies.	Conference abstracts, review papers, technical reports, study protocols, letters, or trial registrations; non–English-language studies; preprints.
Publication date	On or before July 31, 2026	—^a^

a Not applicable.

### Information Sources

A literature search was conducted across major databases, including PubMed, Scopus, Embase, Web of Science, and EconLit. The search covered studies published from database inception to July 31, 2026, with the final search run in full on August 12, 2026. A separate search strategy was applied to each database.

### Search

The search was developed and reported in accordance with the PRISMA-S (Preferred Reporting Items for Systematic Reviews and Meta-Analyses literature search extension) guidelines [25]. The strategy was developed around 3 concepts: (1) economic evaluation; (2) AI-assisted technologies; and (3) older adults and aging-related populations, conditions, and care settings. A broad range of terms was combined using “OR” within each concept, and the 3 concept blocks were combined using “AND.”

Where supported by the database, controlled vocabulary terms, including MeSH and Emtree headings, were combined with free-text synonyms, acronyms, spelling variants, phrases, and truncated terms. Economic evaluation terms covered different forms and outcomes of economic evaluation, including cost-effectiveness, cost-utility, cost-benefit, cost-minimization, cost-consequence, economic modeling, incremental cost-effectiveness ratios (ICERs), and pharmacoeconomics. AI-related terms included AI, machine learning, deep learning, neural networks, natural language processing, computer vision, expert systems, clinical decision-support systems, computer-assisted diagnosis and detection, and automated screening and classification. The population block included direct age-related terms as well as terms describing aging-related conditions and settings, such as frailty, dementia, Alzheimer’s disease, mild cognitive impairment, nursing homes, and care homes. These proxy terms were included because potentially relevant studies may describe the condition or care setting without explicitly referring to older adults. Publication-type and language filters were applied during the database searches.

The strategy was translated according to the controlled vocabulary, field codes, truncation rules, and syntax of each database. Following refinement, the final strategy was run in full and separately in all 5 databases on August 12, 2026, covering database inception through July 31, 2026. The complete strategies exactly as run, together with the search dates and number of records retrieved, are provided in Table S1 in Multimedia Appendix 1.

### Selection of Sources of Evidence

Two authors (QG and MH) independently screened the titles and abstracts of retrieved records to identify eligible studies. Full-text articles were then assessed against the predefined inclusion criteria. Disagreements during study selection were infrequent (approximately 3% of screening decisions). All discrepant records were reviewed jointly by the 2 reviewers (QG and MH), and consensus was reached through discussion before finalizing study inclusion.

### Data Charting Process

Data charting was conducted by 2 reviewers (QG and MH) using a standardized form developed in Microsoft Excel and piloted on a subset of included studies (n=3) to ensure consistency and completeness. The data charting form was adapted from a recent review on economic evaluations of AI interventions in health care [15]. Any discrepancies in the extracted data were resolved through discussion until consensus was reached.

### Data Items

For each included study, information was systematically extracted across 5 domains: study characteristics, study setting, technology characteristics, methodology, and results.

With respect to study characteristics, extracted information included the study title, authors, journal of publication, and publication year. Data on study setting encompassed the target population (including gender focus and age range), intervention context, targeted disease or condition, and study design. Technology characteristics included the name of the intervention and the functional role of AI within the intervention. Methodological information captured comprised the type of economic evaluation, analytical perspective, comparator, and health outcome measures. In addition, detailed AI-related cost information was extracted to elucidate how such costs were identified and calculated. This included the average AI-related cost per patient or per procedure, the composition of AI-related cost components, and the data sources used to derive these estimates. Finally, outcome data extracted from the results sections included incremental health outcomes, incremental costs, principal summary measures (eg, ICER or incremental cost–utility ratio, where applicable), findings from subgroup analyses, and authors’ conclusions regarding whether the intervention was cost-effective or cost-saving.

### The Use of ICOPE Care Pathway

Although the ICOPE framework is primarily designed for primary care settings [4], it offers a coherent and comprehensive structure for conceptualizing the continuum of integrated care for older populations, spanning screening, formal diagnosis, treatment, and follow-up. We therefore adopted the ICOPE stages as an organizing framework to enable consistent stratification and comparison of our included studies.

In reporting the methodological findings of the included studies, AI interventions were classified according to the ICOPE care pathway—basic assessment, in-depth assessment, personalized care planning, and implementation or monitoring—based on their primary function within the care continuum. Specifically, basic screening and assessment refers to initial, broad-based evaluations designed to identify individuals at risk, whereas in-depth assessment involves more comprehensive and multidimensional evaluations to confirm conditions, determine severity, and inform subsequent care planning. While this framework facilitates a standardized synthesis and comparison of evidence across the continuum of older adult care, we acknowledge that some interventions may not fit neatly within a single ICOPE stage, and the classification may not fully capture technologies with cross-cutting functions or broader health system impacts. For interventions spanning multiple stages, classification reflected the dominant role of the AI tool’s intended use. However, when an intervention was explicitly designed to support multiple stages of the care pathway, it was classified as spanning multiple ICOPE stages rather than being assigned to a single category.

### Critical Appraisal of Individual Sources of Evidence

To check the quality and reliability of included studies, 2 reviewers (QG and MH) independently assessed all included studies (n=40) [26-65] using the Criteria for Health Economic Quality Evaluation (CHEQUE) [66]. CHEQUE is a structured appraisal tool developed to assess the methodological rigor and reporting quality of full economic evaluations in health care. The checklist covers key aspects of economic evaluation, including study design, analytical methods, costing, outcome measurement, uncertainty analysis, transparency of reporting, and whether broader considerations, such as equity and ethical issues, were appropriately addressed. For each included study, 24 items assessing methodological rigor and 24 items assessing reporting quality (48 items in total) were evaluated. Each item was rated using a categorical scoring system to indicate whether the corresponding criterion was adequately met. Each of the 24 items in the CHEQUE tool is assigned a precalibrated importance score, derived from a best-worst scaling survey, with weights summing to 100 across items [66]. Each item’s importance score is then multiplied by a credit weight corresponding to its rating: “yes” receives full credit (1.0), “somewhat” receives half credit (0.5), “no” receives no credit (0), and “N/A” is assigned, at the assessor’s discretion, either full credit or exclusion from the total. The resulting weighted values are summed across all 24 items to yield a final score on a 0-to-100 scale. Any discrepancies in the assessment were resolved through discussion until consensus was reached. Larger discrepancies were reviewed item by item to ensure consistent interpretation of the assessment criteria.

### Synthesis of Results

Descriptive statistics were used to examine time trends and the distribution of publications. Narrative synthesis was conducted to summarize the results. In terms of targeted illness, conditions were categorized into cancers, ophthalmologic conditions, musculoskeletal conditions, cardiovascular conditions, and others. Categorical variables, including targeted illness, application field, type of methodology, and cost-effectiveness (eg, cost-saving, cost-effective, and not cost-effective), were summarized using frequency counts and percentages. An intervention was classified as cost-saving when it was less costly and more effective than the comparator (ie, dominant), based on the joint assessment of incremental costs and incremental outcomes. Partial economic evaluations reporting cost savings without a formal cost-effectiveness analysis (CEA) were also included in this category. Interventions were classified as cost-effective when they were not classified as cost-saving but met the study-specific willingness-to-pay threshold. This included interventions that were more costly and more effective with an ICER below the willingness-to-pay threshold, as well as interventions that were less costly and less effective with an ICER above the willingness-to-pay threshold. Interventions that did not meet these criteria, including dominated interventions (ie, more costly and less effective), were classified as not cost-effective. Although cost-saving interventions are also considered cost-effective, we intentionally distinguished these 2 concepts in this study to better illustrate the value for money.

To facilitate comparability among studies, reported costs were adjusted to 2024 price levels and converted to US dollars. Average annual exchange rates from the US Internal Revenue Service website (Internal Revenue Service, U.S. Department of the Treasury) were used for conversion based on average historical exchange rate data for the year 2024. Study quality and methodological rigor, as assessed using the CHEQUE checklist [66], were used to contextualize the findings and inform the interpretation of the evidence during the narrative synthesis. No formal quality-based weighting or exclusion of studies was applied. Visual representations, including bar charts, geographic maps, gap maps, and summary tables, were generated to support the synthesis and provide a clear overview of study characteristics and outcomes. A sensitivity analysis was conducted using the conventional definition of older adults (age≥65 years). A formal assessment of reporting bias was not conducted because the included studies were highly heterogeneous in terms of AI interventions, study designs, economic evaluation methods, and outcome measures, precluding quantitative synthesis. Consequently, publication bias was considered qualitatively during the interpretation of the findings and discussed as a limitation of the review.

To identify the key factors influencing the cost-effectiveness of AI interventions, we synthesized factors reported by the included studies as influencing economic outcomes. These determinants were extracted from sensitivity analyses (eg, one-way, probabilistic, or threshold analyses) and the authors’ interpretation of the primary drivers of cost-effectiveness. Factors that were consistently reported across multiple studies or shown to have a substantial influence on cost-effectiveness outcomes were summarized narratively. Owing to the heterogeneity of study designs, AI interventions, and economic evaluation methods, no quantitative ranking or meta-analysis of determinant importance was performed.

## Results

### Selection of Sources of Evidence

The final database search identified 6512 records across PubMed, Scopus, Embase, Web of Science, and EconLit. After removing 1434 duplicates using the Rayyan platform (Rayyan Systems Inc), 5078 studies proceeded to title and abstract screening, of which 5001 were excluded for the following reasons: not an economic evaluation (n=4436); not focused on AI-related interventions (n=415); not focused on older population (n=48); not conducted for health care purposes (n=12); and review papers (n=90). Two additional studies were excluded due to unavailability of the full text. A total of 75 articles underwent full-text review, resulting in 35 further exclusions (not an economic evaluation: n=3; not focused on older populations: n=11; and not focused on AI-related interventions: n=21). Ultimately, 40 studies met the inclusion criteria for results synthesis (Figure 1) [26-65]. Studies were excluded as “not economic evaluations” if they did not report a full or partial economic evaluation. Full economic evaluations included studies comparing both costs and health outcomes of 2 or more alternatives (eg, cost-effectiveness, cost-utility, cost-benefit, or cost-minimization analyses), whereas partial economic evaluations included studies reporting costs, resource use, or cost analyses. Studies were excluded as “not AI-related interventions” if the intervention did not involve AI technologies according to the predefined eligibility criteria.

**Figure 1.**
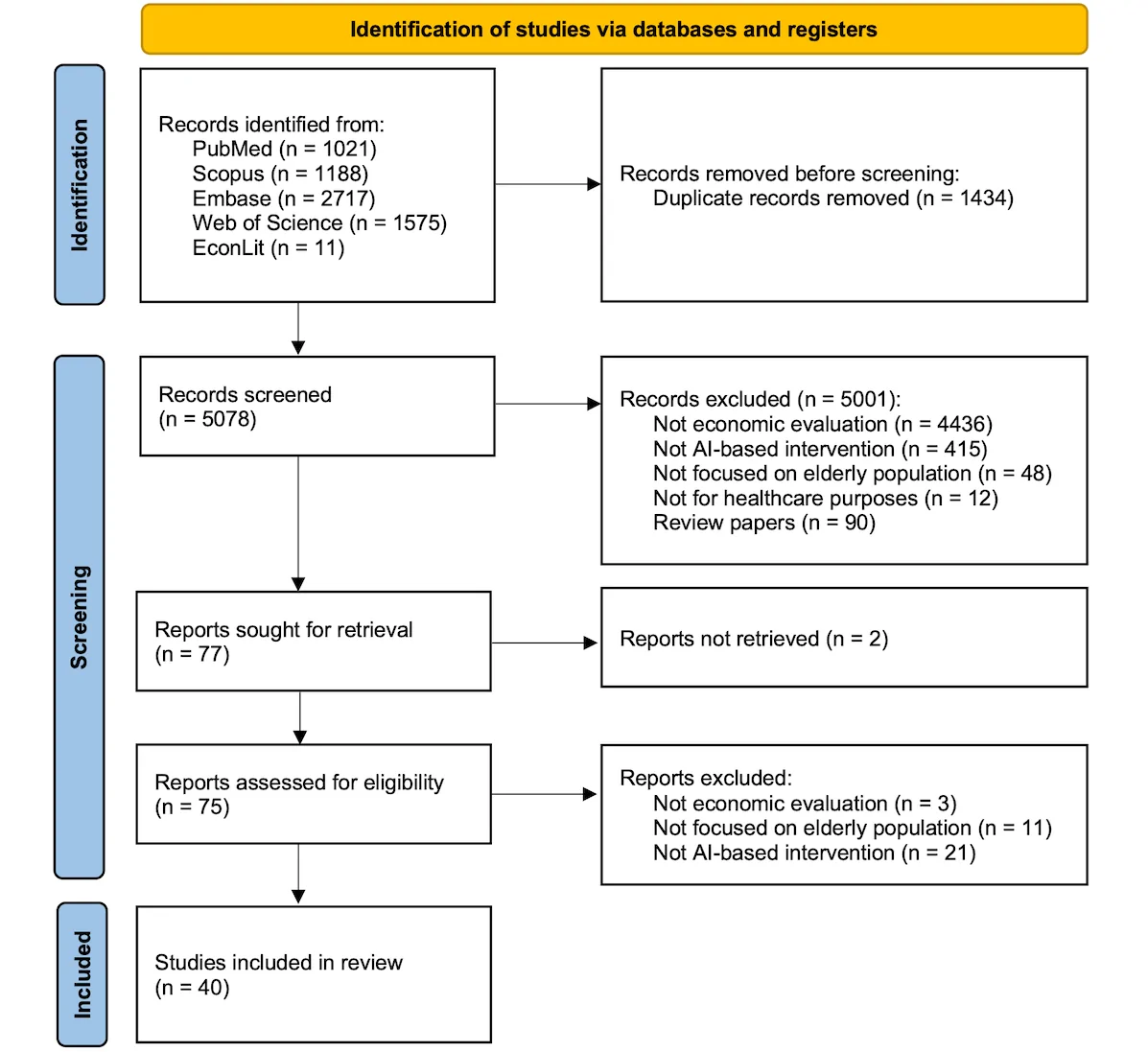
PRISMA (Preferred Reporting Items for Systematic Reviews and Meta-Analyses) flow diagram. This diagram displays the screening process of the scoping review in accordance with the PRISMA-ScR (Preferred Reporting Items for Systematic Reviews and Meta-Analyses extension for Scoping Reviews) guidelines.

### Characteristics of Sources of Evidence

Tables S5 and S6 in Multimedia Appendix 1 provide a detailed summary of study characteristics. The distributions of publications are presented in Figure 2. The earliest included evaluation was published in 2018, examining the cost-effectiveness of PARO, a socially assistive robotic seal used for dementia care [26]. A notable surge in publications occurred from 2021 onwards. The studies were conducted across 16 contexts, reflecting a broad geographical spread, with the United States being the most frequently studied context. Most studies included community or general-population samples (n=23) [27,29-31,34-39,42-44,46,49-51,54,57,58,60,64,65], while 14 focused on older adults with specific conditions [26,28,32,33,40,41,45,48,52,53,56,61-63]. Three studies investigated specific subgroups, including smokers [47], socioeconomically marginalized populations [55], and rural residents [59]. Cancer was the most frequently studied disease area (n=17) [29,30,33,35,37,45-47,51,55-58,60,61,63,64]. Other commonly examined conditions included cardiovascular conditions (n=6) [27,28,39,42,48,59], ophthalmologic diseases (n=6) [31,34,36,43,53,54], and musculoskeletal conditions (n=6) [38,40,49,50,62,65].

**Figure 2.**
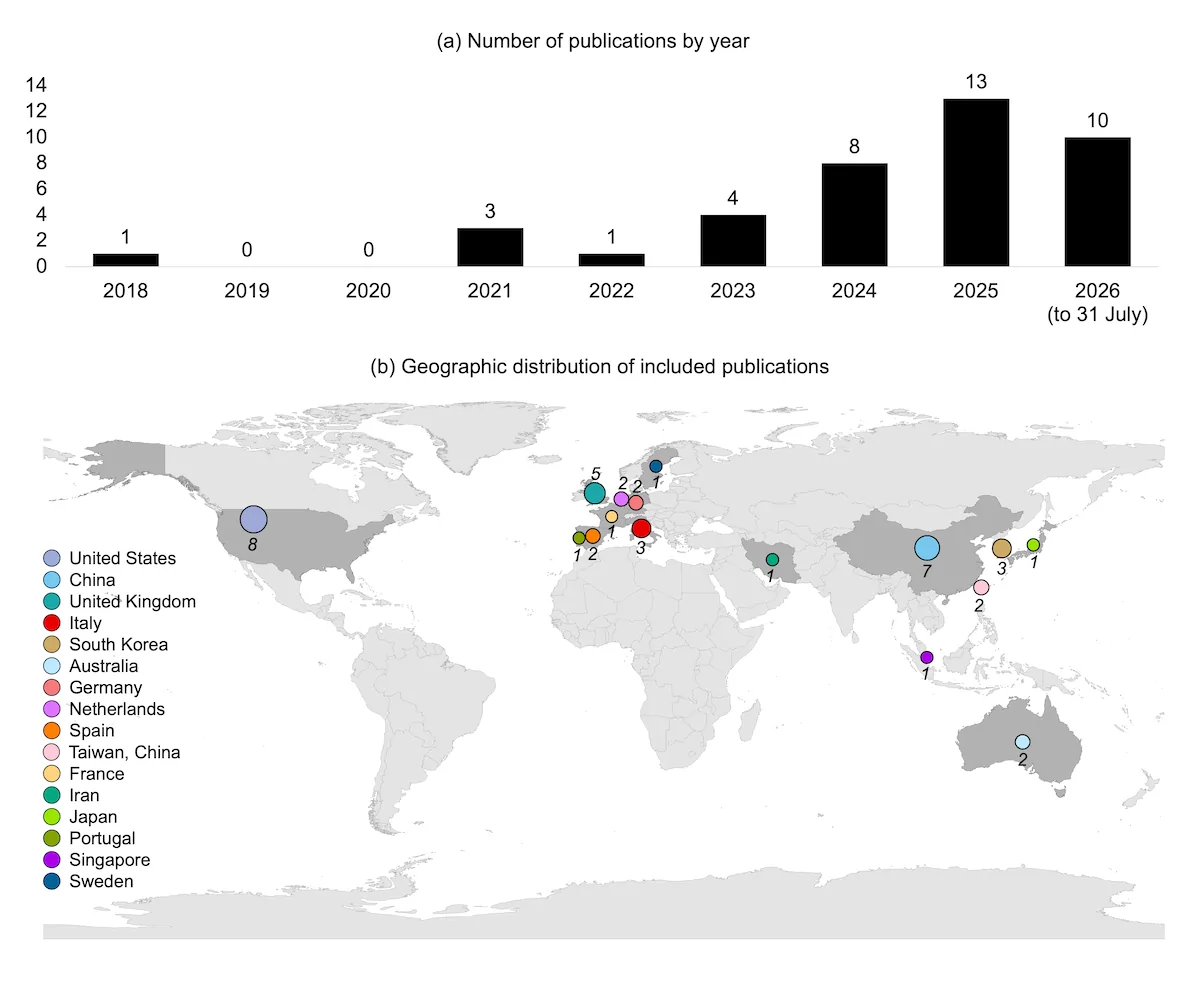
Number of included economic evaluation studies by publication year and by geographic setting. Contexts were classified according to the study setting and health care system reported in the original publication. Multicontext studies contributed to multiple context-specific counts, and the sum of these counts may exceed the total number of included studies.

### Critical Appraisal Within Sources of Evidence

All included studies were critically appraised using the CHEQUE appraisal tool [66]. The study-level CHEQUE appraisal results are presented in Tables S2-S4 in Multimedia Appendix 1. The included studies demonstrated generally high methodological and reporting standards, with mean methodological quality scores of 85.7/100 (SD 7.3) and reporting quality scores of 85.4/100 (SD 7.8) [26-65]. Interrater reliability between 2 reviewers (QG and MH) was high, with Cohen kappa values of 0.853 (91.9% agreement) for methodological quality assessment and 0.880 (91.7% agreement) for reporting quality assessment. Although quality scores varied across studies, all included studies scored above 60 (ranged from 69 to 96 for methodology and from 64.5 to 98 for reporting quality), indicating generally moderate to high methodological quality with no studies demonstrating poor overall quality. Most studies clearly described the intervention setting, modeling approach, cost and health outcomes, and data sources, indicating relatively strong adherence to core principles of economic evaluation. However, few studies explicitly addressed equity considerations, ethical implications, or subgroup heterogeneity. Additionally, model validation (internal or external) was not reported in 21 of the 40 included studies [28-30,32-34,36-38,40-42,44,46,47,52,56,60,62-64], limiting confidence in the accuracy, credibility, and generalizability of the reported economic outcomes. Without validation, it is difficult to assess whether model predictions accurately represent real-world clinical and economic outcomes or can be reliably extrapolated to other settings and populations. Consequently, estimates of costs, health outcomes, and cost-effectiveness may be subject to considerable uncertainty, reducing confidence in the evidence used to inform health care decision-making and policy.

### Synthesis of Results

As shown in Figure 3, most evaluations for cancers, ophthalmologic diseases, and cardiovascular conditions were concentrated on Step 1 (n=24), which focuses on basic screening and assessment [27,29-31,34-39,42,43,47,49-51,54,55,57-60,64,65]. In this stage, AI primarily supports diagnostic judgment and early risk identification by analyzing medical images or other data with computer vision and predictive algorithms, facilitating more efficient detection of potential declines in intrinsic capacity. Eight evaluations addressed Step 2 [28,33,41,45,46,48,61,62], where AI aids in in-depth clinical assessment and formal diagnosis using similar functions as in Step 1; however, these applications remain largely clinically oriented and rarely consider broader factors such as social or environmental determinants highlighted in the ICOPE framework [4].

**Figure 3.**
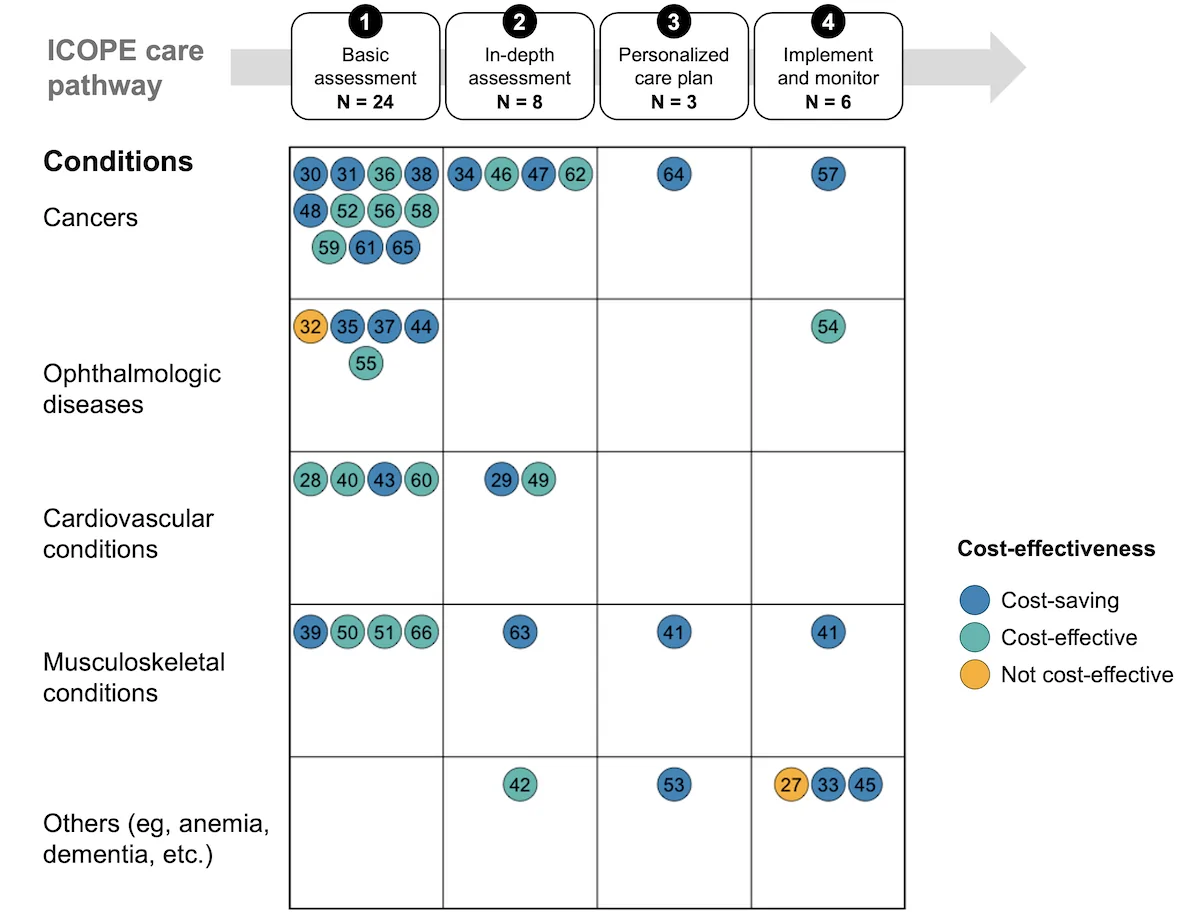
Distribution of included studies by targeted health condition and cost-effectiveness, mapped across the ICOPE care pathway. ICOPE: integrated care for older people.

Evidence for later stages of the pathway remains limited. Only 3 evaluations examined Step 3 [40,52,63], where AI supports the development of personalized care planning through data-driven risk stratification and decision support. Six studies addressed Step 4 [26,32,40,44,53,56], demonstrating the potential of AI-enabled monitoring systems to track patients’ conditions over time, promote adherence, and dynamically adapt care strategies during long-term management. In Step 4, AI applications reflected a preventive and surveillance-oriented application paradigm, encompassing more geriatric-specific outcomes such as dementia [26], injurious falls [32], and emergency hospitalizations [44].

Overall, while AI shows clear value in strengthening early detection and clinical assessment, only 1 included intervention was identified as spanning multiple stages of the ICOPE care pathway. Applications capable of integrating multiple stages of the ICOPE pathway, particularly linking assessment with personalized care planning, community-based services, and social or caregiver support, remain scarce [40].

### Methodology of Evaluation

Economic evaluation methods differed across stages of the ICOPE pathway, reflecting the distinct characteristics of assessment versus care delivery interventions. For technologies used in Step 1 basic and Step 2 in-depth assessment (n=32), cost-utility analysis (CUA) was the predominant approach (n=26) [27,28,31,33-39,42,43,45-51,54,57,59,60,62,64,65], followed by CEA (n=8) [30,31,34,36,41,55,58,61], with 3 studies reporting both CEA and CUA outcomes [31,34,36]. One study conducted only a cost analysis [29]. Most evaluations relied on model-based methods (96.9%, n=31), including Markov models (n=28) [27,28,30,31,33-36,38,39,42,43,45-51,54,57-62,64,65], decision trees (n=13) [27,29,38,39,41,42,45,47-51,54], and discrete-event simulation [37], with only 1 trial-based study using descriptive comparisons [55]. This predominance of modeling reflects the nature of screening and diagnostic interventions, where benefits arise through earlier detection or improved risk stratification and therefore require extrapolation of long-term health and economic outcomes beyond observed data.

In contrast, for interventions used in personalized care planning, implementation, and long-term monitoring (n=8), trial-based evaluations were more common (n=5) [26,32,44,53,56], as these technologies are typically embedded within ongoing care processes and generate observable short- to medium-term outcomes, such as changes in disease control, health care usage, or quality of life, that can be directly measured within defined follow-up periods. However, CUA and model-based approaches were still applied (n=3) to capture potential long-term health gains associated with individualized care planning [40,52,63]. Overall, the evidence shows a methodological shift along the pathway: model-based evaluations dominate early assessment stages, whereas trial-based evaluations are more common in later care implementation and monitoring stages where outcomes can be directly observed.

### Perspective and AI Cost

For Step 1 basic and Step 2 in-depth assessment interventions (n=32), studies adopted a wide range of perspectives, including societal (n=13) [28,30,31,34-36,38,41,43,50,54,55,58], health care payer (n=10) [29,37,39,42,46,48,49,59,61,65], national health system (n=6) [33,47,57,60,62,64], and health sector (n=3) [27,45,51]. The societal perspective had the broadest scope, encompassing direct costs (eg, consultations, hospitalizations, medications, plus nonmedical costs like transportation and informal care) and indirect costs, such as lost productivity and caregiver time. In contrast, the health care payer and health system perspectives were limited to direct medical costs covered by the publicly funded system. The health sector perspective resembled the payer perspective but included all direct health care costs, regardless of funding source. For interventions in Step 3 personalized care planning and Step 4 implementation and long-term monitoring, perspectives were more uniform: six of eight studies adopted the health care payer perspective [26,40,44,52,53,63], one adopted the national health system perspective [56], and one adopted the health sector perspective [32].

For the evaluation of assessment-focused technologies (n=32), cost estimation methods varied considerably due to limited real-world implementation data. Only 18.8% of assessment-focused studies used trial-based estimates derived from observed resource use (n=6) [31,34,36,41,43,64], while others relied on published literature [28,47,51], manufacturer information [30], expert consultation [38,45,51,54,60], or assumptions [27,33,35,38,39,42,45,46,48-51,58-60,62,65]. Some studies approximated AI costs using the price of comparable conventional procedures or by averaging prices of existing AI tools available on the market [30,35]. Reported per-patient or per-procedure AI costs for machine learning–based systems typically ranged from US $0 to $20, although a few technologies exceeded US $100 [39,45,48,61]. Three studies reported AI-related costs of US $0. In 1 study, AI-related costs were excluded in the economic analysis [29], whereas the other 2 assumed zero AI-related cost because the AI platform was open source or the platform cost was considered negligible and therefore omitted [37,58]. In contrast, AI cost estimation in care planning and implementation studies was generally more empirically grounded, with 4 evaluations using trial data [26,32,40,53], allowing AI-related costs to be obtained directly from trials or manufacturer data, typically ranging from US $0 to US $24.6 per patient. Gandjour et al [52] reported AI-related costs as US $0 because they were assumed to be out-of-pocket expenses incurred by patients, and the evaluation was conducted from a health care payer perspective. These studies also provided clearer descriptions of the cost structure of AI interventions, distinguishing between implementation costs (eg, algorithm development, model training, and validation) and operational costs (eg, maintenance, software updates, technical support, and administrative oversight), alongside common cost components such as hardware, software licensing, personnel time, and institutional overheads [53].

### Cost-Effectiveness Results

Overall, the majority of the included AI interventions demonstrated favorable potential in improving health care outcomes and reducing costs. Specifically, 52.5 % of the included studies (n=21) reported their interventions as cost-saving. This included 20 full economic evaluations in which the AI intervention was both less costly and more effective than the comparator (ie, dominant) [28,30,32-34,36-38,40,42-44,46,47,52,56,60,62-64], together with 1 partial economic evaluation that reported cost savings only [29]. Seventeen studies found AI interventions to be cost-effective under the setting-specific willingness-to-pay thresholds used by the authors [27,35,39,41,45,48-51,53-55,57-59,61,65]. No systematic variation in cost-effectiveness by ICOPE stage was apparent, as most interventions are cost-saving or cost-effective at every stage (Figure 3); instead, it is primarily influenced by intervention-specific and contextual factors rather than pathway position. Notably, even AI interventions with relatively high cost (above US $100) yielded cost-effective or even cost-saving conclusions [39,45,48,61,63].

Cost savings were primarily driven by AI’s ability to substitute for or augment human labor in routine tasks, particularly in image interpretation, remote monitoring, and disease surveillance [15,31,34]. However, in settings where labor costs are relatively low, the economic advantage of AI diminishes, as the potential for cost substitution is limited [31]. Improvements in health outcomes were largely attributable to enhanced diagnostic accuracy, earlier disease detection, and more timely initiation of interventions. In addition, AI-enabled risk stratification facilitated more efficient resource allocation by identifying high-risk individuals most likely to benefit from intervention [47].

Only 2 evaluations reported their AI interventions as not cost-effective [26,31], while the underlying drivers differed substantially. Mervin et al [26] evaluated the cost-effectiveness of an AI-assisted robot compared with a plush toy without AI in dementia care; the intervention was associated with relatively high incremental costs and only marginal health gains, resulting in an unfavorable cost-effectiveness result. In Lin et al [31], the lack of cost-effectiveness of the AI-based screening for diabetic retinopathy compared with manual grading appeared to be driven by contextual factors, particularly low labor costs, which reduced the relative advantage of AI-based screening in replacing human labor.

Moreover, several studies have mentioned that AI technologies can improve health equity by increasing the access of advanced imaging tests or other medical services (such as magnetic resonance imaging) in resource-constrained areas [36,45,49,55,59]. However, the equity or distributional considerations of AI were rarely taken into account. One study explicitly raised concerns about implementing AI in rural settings and argued that disparities between and within countries will probably widen [34], while the remainder tended to give optimistic assumptions in terms of the access and uptake of AI.

### Key Factors That Drive Value for Money

This section synthesizes the key determinants of cost-effectiveness for AI technologies in older health care (Figure 4), organized along the ICOPE pathway.

**Figure 4.**
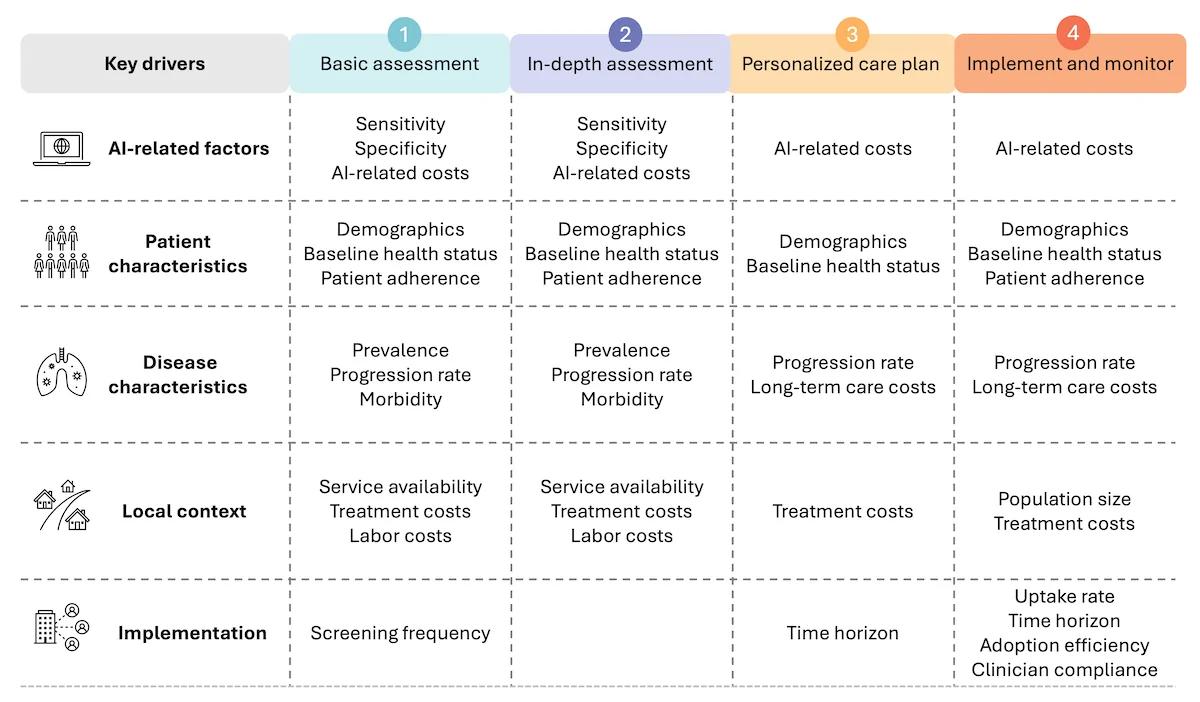
Key drivers of value for money identified across included economic evaluation studies, mapped across the 4 stages of the ICOPE care pathway. ICOPE: integrated care for older people.

For AI-assisted screening and diagnostics, cost-effectiveness is primarily driven by algorithm performance (sensitivity and specificity), AI-related costs, and underlying disease epidemiology [27,29,35,43,45,51]. Higher sensitivity enables earlier detection and treatment, generating quality-adjusted life year (QALY) gains and avoiding downstream complications, particularly in high-prevalence or high-mortality conditions. Furthermore, patient adherence and engagement further modulate outcomes, as improved uptake and persistence can substantially enhance both health gains and cost offsets [31,43,49]. Structural design parameters also shape economic outcomes. Screening frequency determines the trade-off between program costs and incremental health benefits, while the availability and effectiveness of subsequent treatments govern whether improved detection translates into meaningful gains [28,34,36,37,39]. Factors such as age, multimorbidity burden, and baseline health status influenced both achievable health gains and downstream health care usage [27,34,36,39,41,49].

For AI technologies supporting individualized care strategies and disease management, cost-effectiveness was shaped primarily by patient characteristics, adoption efficiency, local facility settings, and long-term disease trajectories [26,40,53]. Specifically, adoption efficiency captures the extent to which AI tools are integrated into routine workflows without generating excessive transaction costs. Key parameters reflecting adoption efficiency include clinician uptake, training requirements, interoperability with electronic health records, alert fatigue, and algorithm update cycles.

Overall, patient complexity and contextual factors emerged as important determinants of the economic value of AI interventions in older health care. Multimorbidity, frailty, and functional status may interact with AI performance in complex ways, while implementation factors such as digital literacy, usability, and care coordination were frequently identified as influencing whether AI interventions achieved health gains at acceptable cost [15].

### Sensitivity Analysis

Sensitivity analysis restricted to studies involving populations aged ≥65 years yielded findings consistent with the primary analysis. Specifically, the included studies remained distributed across the entire ICOPE pathway, although more studies from Steps 1 and 2 were excluded, suggesting the broader applicability of assessment tools at these stages. The diversity of target conditions and the overall findings on cost-effectiveness remained largely unchanged. Among the 14 included studies, 7 focused on screening or diagnosis [27,28,31,38,42,48,59] and 7 on personalized care planning, monitoring, and management [26,32,40,44,52,53,63]. Target conditions were broadly distributed across ophthalmologic diseases [31,53], cardiac diseases [27,42,48,59], musculoskeletal conditions [38,40], cancers [63], and others [26,32,44,52], with 85.7% (n=12) reported as cost-saving or cost-effective [27,28,32,38,40,42,44,48,52,53,59,63]. Despite differences in analytical methods, the findings consistently indicated that AI performance (eg, diagnostic accuracy), disease epidemiology, implementation costs, health care resource usage, and intervention uptake were among the most influential determinants of cost-effectiveness. In most studies, the base-case conclusions remained robust across plausible parameter ranges, although cost-effectiveness was sensitive to assumptions regarding these key inputs.

## Discussion

### Summary of Evidence

This scoping review synthesized evidence from 40 economic evaluations of AI-assisted technologies mapped across the ICOPE care pathway for older adults. Evaluations were unevenly distributed across the care pathway. AI interventions in older health care are frequently reported to be cost-effective, with many also reported to be cost-saving compared with standard care. Across the included studies, the value-for-money of AI interventions was most commonly driven by algorithm performance, AI-related costs, underlying disease epidemiology, patient characteristics, adoption efficiency, local facility settings, and long-term disease trajectories. These drivers were inferred from deterministic or probabilistic sensitivity analyses and scenario analyses, rather than being directly demonstrated through observed implementation outcomes.

The findings broadly align with prior reviews of evaluations for AI in general populations, which similarly report a predominance of favorable cost-effectiveness findings across disease areas [15,16,67]. Although real-world implementation data remain limited in this emerging field, many included studies relied on modeling assumptions, literature-derived estimates, or expert consultation to estimate model parameters. Such approaches are common in economic evaluations and can strengthen the validity of analyses when the assumptions are appropriate, transparently reported, and supported by credible evidence. However, insufficient reporting of the rationale for assumptions, the selection of experts, or the methods used to elicit expert opinion limited assessment of their validity in some studies. Consequently, while the overall evidence is encouraging, decision-makers should critically evaluate whether the modeling assumptions, expert inputs, and cost estimates are applicable to their own health care setting before using these findings to inform policy or reimbursement decisions. AI interventions may fail to be cost-effective for at least three distinct reasons: (1) high implementation costs with limited incremental benefit, (2) cost reductions offset by significant declines in health outcomes, and (3) context-specific economic conditions, such as low labor costs, that reduce the relative advantage of automation [26,31,35].

Consistent with the wider literature, these conclusions should be interpreted cautiously given heterogeneity in study design, perspective, and cost inclusion [15,31,67]. For example, AI tools designed to support early detection may be cost-effective in high-risk older populations but yield limited value in low-prevalence settings [15]. Similarly, interventions implemented in resource-constrained systems may face higher opportunity costs or scalability barriers [31]. These findings highlight the need for closer alignment between technology design, clinical workflows, and health system constraints when assessing the economic value of AI in older care.

A dominant pattern in the evidence base is the concentration of AI applications in screening and diagnostic pathways, especially for cancers and ophthalmologic conditions. This mirrors findings from earlier reviews, which highlight that economic evaluations of AI are disproportionately focused on early diagnostic settings, where improvements in sensitivity and specificity can translate more directly into downstream cost savings and health gains [15]. In contrast, evidence for AI in chronic disease management or long-term older care, such as dementia or frailty, is limited despite their high burden. Another review proposed AI’s potential to reduce costs in dementia care, but formal economic evaluations are lacking [68].

Although previous literature has emphasized equity as a critical consideration in the implementation of AI technologies in health care [69], few studies included in this review explicitly addressed equity in the context of older health care. Among the included studies, Huang et al [55] and Liu et al [59] highlighted the potential of AI technologies to improve access to care in underserved and rural settings, whereas broader equity considerations were largely absent. In practice, the effectiveness of AI-enabled interventions depends on digital infrastructure, user engagement, and access to health care services, all of which vary substantially across socioeconomic groups [70]. Consequently, older adults with limited access to technology or lower income may be systematically less able to benefit from such interventions, potentially exacerbating existing health inequalities [71]. Moreover, individual-level characteristics, including education, cognitive status, resistance to technology use, and concerns about trust and data privacy may further mediate the usability and effectiveness of AI tools, reinforcing unequal outcomes even when access is nominally available [72].

### Challenges in Conducting Economic Evaluations for AI Technologies in Older Care

Despite growing interest in AI-assisted interventions for older care, rigorous economic evaluation of these technologies remains methodologically challenging. Our review found that many included studies relied on modeling assumptions, expert opinion, or nonempirical estimates, reflecting the difficulty of obtaining real-world implementation data. Factors identified in the included studies, such as limited digital literacy and inadequate infrastructure, may help explain this evidence gap [59]. The broader literature has also highlighted challenges including human–AI interaction, evolving algorithms, and performance drift over time, all of which may complicate the generation of stable empirical evidence for economic evaluation [73].

Compounding this, AI interventions present distinct challenges for HTA that are not fully captured by conventional economic modeling frameworks. First, dynamic features of AI systems, such as learning curves and performance improvements over time, were rarely considered or modeled, although they play a key role in determining the cost and effectiveness of AI [74]. For example, performance drift may occur when AI models are applied in new populations or settings, potentially reducing effectiveness relative to initial validation studies [74]. In addition, human-AI interaction plays a critical role in determining real-world outcomes, as clinician behavior, trust, and adherence to AI recommendations can significantly influence effectiveness [75]. Finally, workflow integration costs, including training, infrastructure, and changes to care processes, are often substantial but inconsistently captured in economic evaluations [76].

Meanwhile, methodological inconsistency and lack of reporting transparency are recurring criticisms in existing reviews of AI economic evaluations [15,16,67], and our study is no exception. Many evaluations included in this review omitted key details on cost inputs, price year, and model assumptions, limiting their interpretability and reproducibility. Despite the introduction of Consolidated Health Economic Evaluation Reporting Standards for Interventions That Use Artificial Intelligence (CHEERS-AI), a specially designed reporting standard for health economic evaluations of AI technologies, its uptake remains limited, and a unified scoring system is lacking [74,77]. In older care, where resource allocation decisions are often high-stakes and contested [78], this inability to compare findings across studies substantially constrains the capacity of payers and HTA agencies to make well-informed coverage decisions.

Only a minority of studies derived AI-related costs from empirically observed data, whereas many relied on assumptions, expert opinion, or manufacturer-provided information. Although such approaches are often necessary for emerging technologies with limited real-world implementation data, they introduce uncertainty into cost estimates and may affect the robustness and transferability of reported cost-effectiveness results. This highlights the importance of conducting sensitivity analyses and updating economic evaluations as real-world cost data become available. Unlike conventional medical technologies, the costs of AI systems may change substantially over time owing to software updates, licensing arrangements, economies of scale, computational infrastructure, and maintenance requirements. Future economic evaluations should prioritize the collection and incorporation of real-world implementation and maintenance cost data to improve the validity and policy relevance of economic evidence.

### Implications for HTA-Informed Policy

The findings of this review have several important implications for HTA and policy decision-making regarding AI-enabled technologies. While well-established HTA guidelines exist for drugs and pharmaceuticals, the evaluation of AI-enabled technologies remains an emerging and rapidly evolving field. First, as reflected in our review, existing economic evaluation approaches rarely capture the unique characteristics of AI interventions and the interaction with the specific features of older adult populations, including their adaptive nature, dependence on data quality, and interaction with clinical workflows. There is therefore a need to develop or adapt methodological and reporting guidelines specifically for AI. Although CHEERS-AI provides guidance on reporting AI-specific items [74,77], it does not provide methodological guidance on how these characteristics of AI in supporting complex clinical, functional, and care needs of older adults should be incorporated into economic evaluation.

Second, the widespread reliance on modeling assumptions, the limited use of real-world implementation data, and the lack of model validation identified in this review suggest that policymakers should interpret reported cost-effectiveness estimates with appropriate caution. Consistent with broader HTA principles [79], greater transparency in model assumptions, validation procedures, and reporting would improve the decision relevance of AI economic evaluations.

Third, our review identified that equity considerations were infrequently incorporated into economic evaluations. This is particularly concerning in older care, where differences in functional capacity, digital literacy, socioeconomic status, caregiver support, and access to health and social care may influence both the adoption and effectiveness of AI-enabled interventions. Failure to account for these factors may lead to AI technologies that improve average outcomes while inadvertently widening health inequalities among older adults.

Finally, factors such as labor costs, patient adherence, and local service availability were identified by some of the included economic evaluations as key drivers of cost-effectiveness for AI-enabled interventions in older care [40,55]. This suggests that the cost-effectiveness of these technologies is contingent not only on the intervention itself but on local context and infrastructure. We therefore recommend that HTA processes explicitly incorporate implementation-related parameters, including workforce readiness, infrastructure requirements, and integration into care pathways as part of the evidence base for reimbursement or adoption decisions, rather than treating implementation as a downstream operational concern separate from the economic case.

### Limitations

This scoping review has several limitations. First, the number of eligible economic evaluations of AI in older health care was relatively small. Although AI applications in health care are expanding rapidly, only a limited subset of studies met the inclusion criteria for economic evaluation. Moreover, not all included studies explicitly targeted older populations; in some cases, older adults were embedded within broader adult samples and reported as age-specific subgroup results. Although only older group results were extracted and reported in this review, this may still limit the extent to which the findings fully capture age-specific cost structures, care needs, and outcome trajectories. However, sensitivity analyses restricted to studies involving participants aged ≥65 years yielded findings consistent with the primary analysis, suggesting that this broader inclusion criterion did not materially influence the overall conclusions. Second, the existing evidence base is heavily skewed toward AI applications in screening and diagnosis, and provides limited insights into the economic value of AI technologies used in long-term care, chronic disease management, or socially interactive support interventions. Third, few studies validated their modeled estimates against actual implementation experience. Consequently, the cost-effectiveness patterns identified in this review largely reflect projected rather than empirically confirmed outcomes, and cross-study comparisons may be influenced by differences in modeling assumptions rather than convergent real-world evidence. This limits our ability to draw conclusions about the real-world cost-effectiveness and implementation performance of AI interventions in older care. Finally, given that most included studies reported favorable cost-effectiveness results, publication bias and selective evaluation cannot be ruled out. Studies with null or negative findings may be less likely to be published, and economic evaluations may preferentially focus on AI interventions with strong anticipated clinical or economic performance. Together, these factors may contribute to an overestimation of the value of AI interventions in older health care. Although this review was not prospectively registered in PROSPERO (International Prospective Register of Systematic Reviews) or Open Science Framework (OSF), which may limit transparency regarding deviations from the planned methods, it was conducted in accordance with established methodological guidance and reporting standards for scoping reviews, including the JBI methodology and the PRISMA-ScR guidelines, to promote methodological rigor, transparency, and reproducibility.

### Conclusions

In conclusion, this review finds that the majority of AI-related interventions in older health care were reported to be cost-effective and even cost-saving compared with standard care. However, these findings are based on heterogeneous economic evaluations, many of which relied on modeling assumptions, expert opinion, or limited empirical implementation data, and should therefore be interpreted with appropriate caution. Moreover, the evidence base remains limited in scope, with the majority of included studies focusing on screening and diagnostic interventions. By systematically mapping these gaps and identifying the key drivers of cost-effectiveness across applications, this review contributes an aging-specific lens that is largely absent from the broader AI health-economics literature, and lays the groundwork for methodological standards tailored to this population. In practice, these findings can inform how HTA agencies and policymakers, particularly in universal health coverage systems, incorporate affordability, feasibility, and equity alongside cost-effectiveness when making coverage and scale-up decisions for AI in older care. To inform policy and practice, future research should improve transparency and consistency in reporting, and adopt standardized checklists for AI-related economic evaluations to enhance comparability and credibility.

## Supplementary material

10.2196/104019 Multimedia Appendix 1 Search strategies, quality assessments, study characteristics, and sensitivity analysis.

10.2196/104019 Checklist 1 PRISMA-ScR checklist.

## Abbreviations

CEA: cost-effectiveness analysis
CHEERS-AI: Consolidated Health Economic Evaluation Reporting Standards for Interventions That Use Artificial Intelligence
CHEQUE: Criteria for Health Economic Quality Evaluation
CUA: cost-utility analysis
DHI: digital health intervention
HTA: health technology assessment
ICER: Incremental Cost-Effectiveness Ratio
ICOPE: integrated care for older people
JBI: Joanna Briggs Institute
OSF: Open Science Framework
PRISMA-S: Preferred Reporting Items for Systematic Reviews and Meta-Analyses literature search extension
PRISMA-ScR: Preferred Reporting Items for Systematic Reviews and Meta-Analyses extension for Scoping Reviews
PROSPERO: International Prospective Register of Systematic Reviews
QALY: quality-adjusted life year
WHO: World Health Organization
