# Supplementary material for: Assessing the Value for Money of AI-Assisted Technologies for Older Adults: Scoping Review of Economic Evaluations
Source: J Med Internet Res. 2026 Sep 30;28:e104019. doi: 10.2196/104019 (PMC13626397; doi:10.2196/104019)
Supplement: Supplementary file 2 [file jmir-v28-e104019-s001.docx]

**Supplementary Material 1**

**Table S1** Searching strategies by database.

| **Database** | **Searching term** | **Last search date** | **Records retrieved** |
| --- | --- | --- | --- |
| PubMed | ("Costs and Cost Analysis"[Mesh] OR "Cost-Benefit Analysis"[Mesh] OR "Economics, Medical"[Mesh] OR "Models, Economic"[Mesh] OR "Technology Assessment, Biomedical"[Mesh] OR "Quality-Adjusted Life Years"[Mesh] OR "economic evaluation*"[Title/Abstract] OR "economic analys*"[Title/Abstract] OR "economic model*"[Title/Abstract] OR "economic stud*"[Title/Abstract] OR "health economic*"[Title/Abstract] OR "cost effectiv*"[Title/Abstract] OR "cost utilit*"[Title/Abstract] OR "cost benefit*"[Title/Abstract] OR "cost minimi*"[Title/Abstract] OR "cost consequence*"[Title/Abstract] OR "cost analys*"[Title/Abstract] OR "cost evaluat*"[Title/Abstract] OR "cost saving*"[Title/Abstract] OR "budget impact*"[Title/Abstract] OR "budgetary impact*"[Title/Abstract] OR "value for money"[Title/Abstract] OR "return on investment"[Title/Abstract] OR "incremental cost*"[Title/Abstract] OR ICER[Title/Abstract] OR pharmacoeconomic*[Title/Abstract])  AND(  "Artificial Intelligence"[Mesh] OR "Machine Learning"[Mesh] OR "Deep Learning"[Mesh] OR "Neural Networks, Computer"[Mesh] OR "Natural Language Processing"[Mesh] OR "Pattern Recognition, Automated"[Mesh] OR "Diagnosis, Computer-Assisted"[Mesh] OR "Decision Support Systems, Clinical"[Mesh] OR "Expert Systems"[Mesh] OR "artificial intelligence"[Title/Abstract] OR "artificially intelligent"[Title/Abstract] OR "AI assist*"[Title/Abstract] OR "AI based"[Title/Abstract] OR "AI enabled"[Title/Abstract] OR "AI driven"[Title/Abstract] OR "machine learning"[Title/Abstract] OR "deep learning"[Title/Abstract] OR "supervised learning"[Title/Abstract] OR "unsupervised learning"[Title/Abstract] OR "reinforcement learning"[Title/Abstract] OR "transfer learning"[Title/Abstract] OR "federated learning"[Title/Abstract] OR "neural network*"[Title/Abstract] OR "natural language processing"[Title/Abstract] OR NLP[Title/Abstract] OR "computer vision"[Title/Abstract] OR "image recognition"[Title/Abstract] OR "pattern recognition"[Title/Abstract] OR "expert system*"[Title/Abstract] OR "intelligent system*"[Title/Abstract] OR "clinical decision support"[Title/Abstract] OR "decision support system*"[Title/Abstract] OR CDSS[Title/Abstract] OR "computer aided diagnos*"[Title/Abstract] OR "computer assisted diagnos*"[Title/Abstract] OR "computer aided detect*"[Title/Abstract] OR "computer assisted detect*"[Title/Abstract] OR "automated diagnos*"[Title/Abstract] OR "automated detect*"[Title/Abstract] OR "automated screen*"[Title/Abstract] OR "automated classif*"[Title/Abstract] OR "automated image analys*"[Title/Abstract] OR "predictive algorithm*"[Title/Abstract] OR "prediction algorithm*"[Title/Abstract] OR "machine learning algorithm*"[Title/Abstract] OR "random forest*"[Title/Abstract] OR "generative artificial intelligence"[Title/Abstract] OR "generative AI"[Title/Abstract] OR "large language model*"[Title/Abstract] OR LLM*[Title/Abstract] OR "foundation model*"[Title/Abstract])  AND(  "Aged"[Mesh] OR "Frail Elderly"[Mesh] OR "Geriatrics"[Mesh] OR "Frailty"[Mesh] OR "Dementia"[Mesh] OR "Alzheimer Disease"[Mesh] OR "Mild Cognitive Impairment"[Mesh] OR "Nursing Homes"[Mesh] OR "Assisted Living Facilities"[Mesh] OR "Long-Term Care"[Mesh] OR elder*[Title/Abstract] OR older*[Title/Abstract] OR aged[Title/Abstract] OR aging[Title/Abstract] OR ageing[Title/Abstract] OR geriatric*[Title/Abstract] OR senior*[Title/Abstract] OR "late life"[Title/Abstract] OR "later life"[Title/Abstract] OR frail*[Title/Abstract] OR dement*[Title/Abstract] OR Alzheimer*[Title/Abstract] OR "mild cognitive impairment"[Title/Abstract] OR "cognitive decline"[Title/Abstract] OR "nursing home*"[Title/Abstract] OR "care home*"[Title/Abstract]) | 12 Aug 2026 | 1,021 |
| Scopus | TITLE-ABS-KEY(  "economic evaluat*" OR "economic analys*" OR "economic model*" OR "economic stud*" OR "health economic*" OR "cost effectiv*" OR "cost utilit*" OR "cost benefit*" OR "cost minimi*" OR "cost consequence*" OR "cost analys*" OR "cost evaluat*" OR "cost saving*" OR "budget impact*" OR "budgetary impact*" OR "value for money" OR "return on investment" OR "incremental cost*" OR ICER OR pharmacoeconomic*) AND TITLE-ABS-KEY( "artificial intelligence" OR "artificially intelligent" OR "AI assist*" OR "AI based" OR "AI enabled" OR "AI driven" OR "machine learning" OR "deep learning" OR "supervised learning" OR "unsupervised learning" OR "reinforcement learning" OR "transfer learning" OR "federated learning" OR "neural network*" OR "natural language processing" OR NLP OR "computer vision" OR "image recognition" OR "pattern recognition" OR "expert system*" OR "intelligent system*" OR "clinical decision support" OR "decision support system*" OR CDSS OR "computer aided diagnos*" OR "computer assisted diagnos*" OR "computer aided detect*" OR "computer assisted detect*" OR "automated diagnos*" OR "automated detect*" OR "automated screen*" OR "automated classif*" OR "automated image analys*" OR "predictive algorithm*" OR "prediction algorithm*" OR "machine learning algorithm*" OR "generative artificial intelligence" OR "generative AI" OR "large language model*" OR LLM* OR "foundation model*")  AND TITLE-ABS-KEY( elder* OR older* OR aged OR aging OR ageing OR geriatric* OR senior* OR "late life" OR "later life" OR frail* OR dement* OR Alzheimer* OR "mild cognitive impairment" OR "cognitive decline" OR "nursing home*" OR "care home*") | 12 Aug 2026 | 1,188 |
| Embase | ('health economics'/exp OR 'economic evaluation'/exp OR 'health care cost'/exp OR 'pharmacoeconomics'/exp OR 'cost effectiveness analysis'/exp OR 'cost utility analysis'/exp OR 'cost benefit analysis'/exp OR (economic* NEAR/2 evaluat*):ti,ab,kw OR (economic* NEAR/2 analy*):ti,ab,kw OR (economic* NEAR/2 model*):ti,ab,kw OR (economic* NEAR/2 stud*):ti,ab,kw OR 'health economic*':ti,ab,kw OR (cost* NEAR/2 effectiv*):ti,ab,kw OR (cost* NEAR/2 utilit*):ti,ab,kw OR (cost* NEAR/2 benefit*):ti,ab,kw OR (cost* NEAR/2 minimi*):ti,ab,kw OR (cost* NEAR/2 consequence*):ti,ab,kw OR (cost* NEAR/2 analy*):ti,ab,kw OR (cost* NEAR/2 evaluat*):ti,ab,kw OR (cost* NEAR/2 saving*):ti,ab,kw OR 'budget impact*':ti,ab,kw OR 'budgetary impact*':ti,ab,kw OR 'value for money':ti,ab,kw OR 'return on investment':ti,ab,kw OR 'incremental cost*':ti,ab,kw OR icer:ti,ab,kw OR pharmacoeconomic*:ti,ab,kw)  AND(  'artificial intelligence'/exp OR 'machine learning'/exp OR 'deep learning'/exp OR 'expert system'/exp OR 'artificial neural network'/exp OR 'natural language processing'/exp OR 'clinical decision support system'/exp OR 'artificial intelligence':ti,ab,kw OR 'artificially intelligent':ti,ab,kw OR 'ai assist*':ti,ab,kw OR 'ai based':ti,ab,kw OR 'ai enabled':ti,ab,kw OR 'ai driven':ti,ab,kw OR 'machine learning':ti,ab,kw OR 'deep learning':ti,ab,kw OR 'supervised learning':ti,ab,kw OR 'unsupervised learning':ti,ab,kw OR 'reinforcement learning':ti,ab,kw OR 'transfer learning':ti,ab,kw OR 'federated learning':ti,ab,kw OR (neural NEAR/1 network*):ti,ab,kw OR 'natural language processing':ti,ab,kw OR nlp:ti,ab,kw OR 'computer vision':ti,ab,kw OR 'image recognition':ti,ab,kw OR 'pattern recognition':ti,ab,kw OR 'expert system*':ti,ab,kw OR 'intelligent system*':ti,ab,kw OR 'clinical decision support':ti,ab,kw OR 'decision support system*':ti,ab,kw OR cdss:ti,ab,kw OR 'computer aided diagnos*':ti,ab,kw OR 'computer assisted diagnos*':ti,ab,kw OR 'computer aided detect*':ti,ab,kw OR 'computer assisted detect*':ti,ab,kw OR 'automated diagnos*':ti,ab,kw OR 'automated detect*':ti,ab,kw OR 'automated screen*':ti,ab,kw OR 'automated classif*':ti,ab,kw OR 'automated image analys*':ti,ab,kw OR 'predictive algorithm*':ti,ab,kw OR 'prediction algorithm*':ti,ab,kw OR 'machine learning algorithm*':ti,ab,kw OR 'generative artificial intelligence':ti,ab,kw OR 'generative ai':ti,ab,kw OR 'large language model*':ti,ab,kw OR llm*:ti,ab,kw OR 'foundation model*':ti,ab,kw)  AND(  'aged'/exp OR 'frail elderly'/exp OR 'geriatrics'/exp OR 'geriatric patient'/exp OR 'frailty'/exp OR 'dementia'/exp OR 'Alzheimer disease'/exp OR 'mild cognitive impairment'/exp OR 'nursing home'/exp OR 'assisted living facility'/exp OR 'long term care'/exp OR elder*:ti,ab,kw OR older*:ti,ab,kw OR aged:ti,ab,kw OR aging:ti,ab,kw OR ageing:ti,ab,kw OR geriatric*:ti,ab,kw OR senior*:ti,ab,kw OR 'late life':ti,ab,kw OR 'later life':ti,ab,kw OR frail*:ti,ab,kw OR dement*:ti,ab,kw OR Alzheimer*:ti,ab,kw OR 'mild cognitive impairment':ti,ab,kw OR 'cognitive decline':ti,ab,kw OR 'nursing home*':ti,ab,kw OR 'care home*':ti,ab,kw) | 12 Aug 2026 | 2,717 |
| Web of Science | TS=(  "economic evaluat*" OR "economic analys*" OR "economic model*" OR "economic stud*" OR "health economic*" OR "cost effectiv*" OR "cost utilit*" OR "cost benefit*" OR "cost minimi*" OR "cost consequence*" OR "cost analys*" OR "cost evaluat*" OR "cost saving*" OR "budget impact*" OR "budgetary impact*" OR "value for money" OR "return on investment" OR "incremental cost*" OR ICER OR pharmacoeconomic* )  AND TS=(  "artificial intelligence" OR "artificially intelligent" OR "AI assist*" OR "AI based" OR "AI enabled" OR "AI driven" OR "machine learning" OR "deep learning" OR "supervised learning" OR "unsupervised learning" OR "reinforcement learning" OR "transfer learning" OR "federated learning" OR "neural network*" OR "natural language processing" OR NLP OR "computer vision" OR "image recognition" OR "pattern recognition" OR "expert system*" OR "intelligent system*" OR "clinical decision support" OR "decision support system*" OR CDSS OR "computer aided diagnos*" OR "computer assisted diagnos*" OR "computer aided detect*" OR "computer assisted detect*" OR "automated diagnos*" OR "automated detect*" OR "automated screen*" OR "automated classif*" OR "automated image analys*" OR "predictive algorithm*" OR "prediction algorithm*" OR "machine learning algorithm*" OR "generative artificial intelligence" OR "generative AI" OR "large language model*" OR LLM* OR "foundation model*" )  AND TS=(  elder* OR older* OR aged OR aging OR ageing OR geriatric* OR senior* OR "late life" OR "later life" OR frail* OR dement* OR Alzheimer* OR "mild cognitive impairment" OR "cognitive decline" OR "nursing home*" OR "care home*") | 12 Aug 2026 | 1,575 |
| EconLit | TX (  "economic evaluat*" OR "economic analys*" OR "economic model*" OR "economic stud*" OR "health economic*" OR "cost effectiv*" OR "cost utilit*" OR "cost benefit*" OR "cost minimi*" OR "cost consequence*" OR "cost analys*" OR "cost evaluat*" OR "cost saving*" OR "budget impact*" OR "budgetary impact*" OR "value for money" OR "return on investment" OR "incremental cost*" OR ICER OR pharmacoeconomic* )  AND TX (  "artificial intelligence" OR "artificially intelligent" OR "AI assist*" OR "AI based" OR "AI enabled" OR "AI driven" OR "machine learning" OR "deep learning" OR "supervised learning" OR "unsupervised learning" OR "reinforcement learning" OR "transfer learning" OR "federated learning" OR "neural network*" OR "natural language processing" OR NLP OR "computer vision" OR "image recognition" OR "pattern recognition" OR "expert system*" OR "intelligent system*" OR "clinical decision support" OR "decision support system*" OR CDSS OR "computer aided diagnos*" OR "computer assisted diagnos*" OR "computer aided detect*" OR "computer assisted detect*" OR "automated diagnos*" OR "automated detect*" OR "automated screen*" OR "automated classif*" OR "automated image analys*" OR "predictive algorithm*" OR "prediction algorithm*" OR "machine learning algorithm*" OR "generative artificial intelligence" OR "generative AI" OR "large language model*" OR LLM* OR "foundation model*" )  AND TX (  elder* OR older* OR aged OR aging OR ageing OR geriatric* OR senior* OR "late life" OR "later life" OR frail* OR dement* OR Alzheimer* OR "mild cognitive impairment" OR "cognitive decline" OR "nursing home*" OR "care home*") | 12 Aug 2026 | 11 |

**Table S2** CHEQUE quality assessment results: Methodological Quality.

|  | M1 | M2 | | M3 | | M4 | | M5 | | M6 | | M7 | | M8 | | M9 | M10 | | M11 | | | M12 | | M13 | | | | M14 | | | | M15 | | | M16 | | | | M17 | | | M18 | | | M19 | | M20 | | M21 | | M22 | | M23 | | M24 | |  |
| --- | --- | --- | --- | --- | --- | --- | --- | --- | --- | --- | --- | --- | --- | --- | --- | --- | --- | --- | --- | --- | --- | --- | --- | --- | --- | --- | --- | --- | --- | --- | --- | --- | --- | --- | --- | --- | --- | --- | --- | --- | --- | --- | --- | --- | --- | --- | --- | --- | --- | --- | --- | --- | --- | --- | --- | --- | --- |
| [1] |  |  | |  | |  | |  | |  | |  | |  | |  |  | |  | | |  | |  | | | |  | | | |  | | |  | | | |  | | |  | | |  | |  | |  | |  | |  | |  | |  |
| [2] |  |  | |  | |  | |  | |  | |  | |  | |  |  | |  | | |  | |  | | | |  | | | |  | | |  | | | |  | | |  | | |  | |  | |  | |  | |  | |  | |  |
| [3] |  |  | |  | |  | |  | |  | |  | |  | |  |  | |  | | |  | |  | | | |  | | | |  | | |  | | | |  | | |  | | |  | |  | |  | |  | |  | |  | |  |
| [4] |  |  | |  | |  | |  | |  | |  | |  | |  |  | |  | | |  | |  | | | |  | | | |  | | |  | | | |  | | |  | | |  | |  | |  | |  | |  | |  | |  |
| [5] |  |  | |  | |  | |  | |  | |  | |  | |  |  | |  | | |  | |  | | | |  | | | |  | | |  | | | |  | | |  | | |  | |  | |  | |  | |  | |  | |  |
| [6] |  |  | |  | |  | |  | |  | |  | |  | |  |  | |  | | |  | |  | | | |  | | | |  | | |  | | | |  | | |  | | |  | |  | |  | |  | |  | |  | |  |
| [7] |  |  | |  | |  | |  | |  | |  | |  | |  |  | |  | | |  | |  | | | |  | | | |  | | |  | | | |  | | |  | | |  | |  | |  | |  | |  | |  | |  |
| [8] |  |  | |  | |  | |  | |  | |  | |  | |  |  | |  | | |  | |  | | | |  | | | |  | | |  | | | |  | | |  | | |  | |  | |  | |  | |  | |  | |  |
| [9] |  |  | |  | |  | |  | |  | |  | |  | |  |  | |  | | |  | |  | | | |  | | | |  | | |  | | | |  | | |  | | |  | |  | |  | |  | |  | |  | |  |
| [10] |  |  | |  | |  | |  | |  | |  | |  | |  |  | |  | | |  | |  | | | |  | | | |  | | |  | | | |  | | |  | | |  | |  | |  | |  | |  | |  | |  |
| [11] |  |  | |  | |  | |  | |  | |  | |  | |  |  | |  | | |  | |  | | | |  | | | |  | | |  | | | |  | | |  | | |  | |  | |  | |  | |  | |  | |  |
| [12] |  |  | |  | |  | |  | |  | |  | |  | |  |  | |  | | |  | |  | | | |  | | | |  | | |  | | | |  | | |  | | |  | |  | |  | |  | |  | |  | |  |
| [13] |  |  | |  | |  | |  | |  | |  | |  | |  |  | |  | | |  | |  | | | |  | | | |  | | |  | | | |  | | |  | | |  | |  | |  | |  | |  | |  | |  |
| [14] |  |  | |  | |  | |  | |  | |  | |  | |  |  | |  | | |  | |  | | | |  | | | |  | | |  | | | |  | | |  | | |  | |  | |  | |  | |  | |  | |  |
| [15] |  |  | |  | |  | |  | |  | |  | |  | |  |  | |  | | |  | |  | | | |  | | | |  | | |  | | | |  | | |  | | |  | |  | |  | |  | |  | |  | |  |
| [16] |  |  | |  | |  | |  | |  | |  | |  | |  |  | |  | | |  | |  | | | |  | | | |  | | |  | | | |  | | |  | | |  | |  | |  | |  | |  | |  | |  |
| [17] |  |  | |  | |  | |  | |  | |  | |  | |  |  | |  | | |  | |  | | | |  | | | |  | | |  | | | |  | | |  | | |  | |  | |  | |  | |  | |  | |  |
| [18] |  |  | |  | |  | |  | |  | |  | |  | |  |  | |  | | |  | |  | | | |  | | | |  | | |  | | | |  | | |  | | |  | |  | |  | |  | |  | |  | |  |
| [19] |  |  | |  | |  | |  | |  | |  | |  | |  |  | |  | | |  | |  | | | |  | | | |  | | |  | | | |  | | |  | | |  | |  | |  | |  | |  | |  | |  |
| [20] |  |  | |  | |  | |  | |  | |  | |  | |  |  | |  | | |  | |  | | | |  | | | |  | | |  | | | |  | | |  | | |  | |  | |  | |  | |  | |  | |  |
| [21] |  |  | |  | |  | |  | |  | |  | |  | |  |  | |  | | |  | |  | | | |  | | | |  | | |  | | | |  | | |  | | |  | |  | |  | |  | |  | |  | |  |
| [22] |  |  | |  | |  | |  | |  | |  | |  | |  |  | |  | | |  | |  | | | |  | | | |  | | |  | | | |  | | |  | | |  | |  | |  | |  | |  | |  | |  |
| [23] |  |  | |  | |  | |  | |  | |  | |  | |  |  | |  | | |  | |  | | | |  | | | |  | | |  | | | |  | | |  | | |  | |  | |  | |  | |  | |  | |  |
| [24] |  |  | |  | |  | |  | |  | |  | |  | |  |  | |  | | |  | |  | | | |  | | | |  | | |  | | | |  | | |  | | |  | |  | |  | |  | |  | |  | |  |
| [25] |  |  | |  | |  | |  | |  | |  | |  | |  |  | |  | | |  | |  | | | |  | | | |  | | |  | | | |  | | |  | | |  | |  | |  | |  | |  | |  | |  |
| [26] |  |  | |  | |  | |  | |  | |  | |  | |  |  | |  | | |  | |  | | | |  | | | |  | | |  | | | |  | | |  | | |  | |  | |  | |  | |  | |  | |  |
| [27] |  |  | |  | |  | |  | |  | |  | |  | |  |  | |  | | |  | |  | | | |  | | | |  | | |  | | | |  | | |  | | |  | |  | |  | |  | |  | |  | |  |
| [28] |  |  | |  | |  | |  | |  | |  | |  | |  |  | |  | | |  | |  | | | |  | | | |  | | |  | | | |  | | |  | | |  | |  | |  | |  | |  | |  | |  |
| [29] |  |  | |  | |  | |  | |  | |  | |  | |  |  | |  | | |  | |  | | | |  | | | |  | | |  | | | |  | | |  | | |  | |  | |  | |  | |  | |  | |  |
| [30] |  |  | |  | |  | |  | |  | |  | |  | |  |  | |  | | |  | |  | | | |  | | | |  | | |  | | | |  | | |  | | |  | |  | |  | |  | |  | |  | |  |
| [31] |  |  | |  | |  | |  | |  | |  | |  | |  |  | |  | | |  | |  | | | |  | | | |  | | |  | | | |  | | |  | | |  | |  | |  | |  | |  | |  | |  |
| [32] |  |  | |  | |  | |  | |  | |  | |  | |  |  | |  | | |  | |  | | | |  | | | |  | | |  | | | |  | | |  | | |  | |  | |  | |  | |  | |  | |  |
| [33] |  |  | |  | |  | |  | |  | |  | |  | |  |  | |  | | |  | |  | | | |  | | | |  | | |  | | | |  | | |  | | |  | |  | |  | |  | |  | |  | |  |
| [34] |  |  | |  | |  | |  | |  | |  | |  | |  |  | |  | | |  | |  | | | |  | | | |  | | |  | | | |  | | |  | | |  | |  | |  | |  | |  | |  | |  |
| [35] |  |  | |  | |  | |  | |  | |  | |  | |  |  | |  | | |  | |  | | | |  | | | |  | | |  | | | |  | | |  | | |  | |  | |  | |  | |  | |  | |  |
| [36] |  |  | |  | |  | |  | |  | |  | |  | |  |  | |  | | |  | |  | | | |  | | | |  | | |  | | | |  | | |  | | |  | |  | |  | |  | |  | |  | |  |
| [37] |  |  | |  | |  | |  | |  | |  | |  | |  |  | |  | | |  | |  | | | |  | | | |  | | |  | | | |  | | |  | | |  | |  | |  | |  | |  | |  | |  |
| [38] |  |  | |  | |  | |  | |  | |  | |  | |  |  | |  | | |  | |  | | | |  | | | |  | | |  | | | |  | | |  | | |  | |  | |  | |  | |  | |  | |  |
| [39] |  |  | |  | |  | |  | |  | |  | |  | |  |  | |  | | |  | |  | | | |  | | | |  | | |  | | | |  | | |  | | |  | |  | |  | |  | |  | |  | |  |
| [40] |  |  | |  | |  | |  | |  | |  | |  | |  |  | |  | | |  | |  | | | |  | | | |  | | |  | | | |  | | |  | | |  | |  | |  | |  | |  | |  | |  |
|  |  | |  | | | |  | |  | | | |  | |  | | |  |  | | | | | |  | |  | | |  | | |  | | |  | |  | | |  | | |  | |  | |  | |  | |  | |  | |  | |
|  |  | | No | |  | | Somewhat | | | |  | | Yes | |  | N/A | | | |  |  | |  | | |  | | |  | |  | | |  | | |  | | |  | | |  | | |  | |  | |  | |  | |  |  |  |  |

**Table S3** CHEQUE quality assessment results: Reporting Quality.

|  | R1 | R2 | | R3 | | R4 | | R5 | | R6 | | R7 | | R8 | | R9 | | R10 | | R11 | | R12 | | R13 | | R14 | | R15 | | R16 | | R17 | | R18 | | R19 | | R20 | | R21 | | R22 | | R23 | | R24 |
| --- | --- | --- | --- | --- | --- | --- | --- | --- | --- | --- | --- | --- | --- | --- | --- | --- | --- | --- | --- | --- | --- | --- | --- | --- | --- | --- | --- | --- | --- | --- | --- | --- | --- | --- | --- | --- | --- | --- | --- | --- | --- | --- | --- | --- | --- | --- |
| [1] |  |  | |  | |  | |  | |  | |  | |  | |  | |  | |  | |  | |  | |  | |  | |  | |  | |  | |  | |  | |  | |  | |  | |  |
| [2] |  |  | |  | |  | |  | |  | |  | |  | |  | |  | |  | |  | |  | |  | |  | |  | |  | |  | |  | |  | |  | |  | |  | |  |
| [3] |  |  | |  | |  | |  | |  | |  | |  | |  | |  | |  | |  | |  | |  | |  | |  | |  | |  | |  | |  | |  | |  | |  | |  |
| [4] |  |  | |  | |  | |  | |  | |  | |  | |  | |  | |  | |  | |  | |  | |  | |  | |  | |  | |  | |  | |  | |  | |  | |  |
| [5] |  |  | |  | |  | |  | |  | |  | |  | |  | |  | |  | |  | |  | |  | |  | |  | |  | |  | |  | |  | |  | |  | |  | |  |
| [6] |  |  | |  | |  | |  | |  | |  | |  | |  | |  | |  | |  | |  | |  | |  | |  | |  | |  | |  | |  | |  | |  | |  | |  |
| [7] |  |  | |  | |  | |  | |  | |  | |  | |  | |  | |  | |  | |  | |  | |  | |  | |  | |  | |  | |  | |  | |  | |  | |  |
| [8] |  |  | |  | |  | |  | |  | |  | |  | |  | |  | |  | |  | |  | |  | |  | |  | |  | |  | |  | |  | |  | |  | |  | |  |
| [9] |  |  | |  | |  | |  | |  | |  | |  | |  | |  | |  | |  | |  | |  | |  | |  | |  | |  | |  | |  | |  | |  | |  | |  |
| [10] |  |  | |  | |  | |  | |  | |  | |  | |  | |  | |  | |  | |  | |  | |  | |  | |  | |  | |  | |  | |  | |  | |  | |  |
| [11] |  |  | |  | |  | |  | |  | |  | |  | |  | |  | |  | |  | |  | |  | |  | |  | |  | |  | |  | |  | |  | |  | |  | |  |
| [12] |  |  | |  | |  | |  | |  | |  | |  | |  | |  | |  | |  | |  | |  | |  | |  | |  | |  | |  | |  | |  | |  | |  | |  |
| [13] |  |  | |  | |  | |  | |  | |  | |  | |  | |  | |  | |  | |  | |  | |  | |  | |  | |  | |  | |  | |  | |  | |  | |  |
| [14] |  |  | |  | |  | |  | |  | |  | |  | |  | |  | |  | |  | |  | |  | |  | |  | |  | |  | |  | |  | |  | |  | |  | |  |
| [15] |  |  | |  | |  | |  | |  | |  | |  | |  | |  | |  | |  | |  | |  | |  | |  | |  | |  | |  | |  | |  | |  | |  | |  |
| [16] |  |  | |  | |  | |  | |  | |  | |  | |  | |  | |  | |  | |  | |  | |  | |  | |  | |  | |  | |  | |  | |  | |  | |  |
| [17] |  |  | |  | |  | |  | |  | |  | |  | |  | |  | |  | |  | |  | |  | |  | |  | |  | |  | |  | |  | |  | |  | |  | |  |
| [18] |  |  | |  | |  | |  | |  | |  | |  | |  | |  | |  | |  | |  | |  | |  | |  | |  | |  | |  | |  | |  | |  | |  | |  |
| [19] |  |  | |  | |  | |  | |  | |  | |  | |  | |  | |  | |  | |  | |  | |  | |  | |  | |  | |  | |  | |  | |  | |  | |  |
| [20] |  |  | |  | |  | |  | |  | |  | |  | |  | |  | |  | |  | |  | |  | |  | |  | |  | |  | |  | |  | |  | |  | |  | |  |
| [21] |  |  | |  | |  | |  | |  | |  | |  | |  | |  | |  | |  | |  | |  | |  | |  | |  | |  | |  | |  | |  | |  | |  | |  |
| [22] |  |  | |  | |  | |  | |  | |  | |  | |  | |  | |  | |  | |  | |  | |  | |  | |  | |  | |  | |  | |  | |  | |  | |  |
| [23] |  |  | |  | |  | |  | |  | |  | |  | |  | |  | |  | |  | |  | |  | |  | |  | |  | |  | |  | |  | |  | |  | |  | |  |
| [24] |  |  | |  | |  | |  | |  | |  | |  | |  | |  | |  | |  | |  | |  | |  | |  | |  | |  | |  | |  | |  | |  | |  | |  |
| [25] |  |  | |  | |  | |  | |  | |  | |  | |  | |  | |  | |  | |  | |  | |  | |  | |  | |  | |  | |  | |  | |  | |  | |  |
| [26] |  |  | |  | |  | |  | |  | |  | |  | |  | |  | |  | |  | |  | |  | |  | |  | |  | |  | |  | |  | |  | |  | |  | |  |
| [27] |  |  | |  | |  | |  | |  | |  | |  | |  | |  | |  | |  | |  | |  | |  | |  | |  | |  | |  | |  | |  | |  | |  | |  |
| [28] |  |  | |  | |  | |  | |  | |  | |  | |  | |  | |  | |  | |  | |  | |  | |  | |  | |  | |  | |  | |  | |  | |  | |  |
| [29] |  |  | |  | |  | |  | |  | |  | |  | |  | |  | |  | |  | |  | |  | |  | |  | |  | |  | |  | |  | |  | |  | |  | |  |
| [30] |  |  | |  | |  | |  | |  | |  | |  | |  | |  | |  | |  | |  | |  | |  | |  | |  | |  | |  | |  | |  | |  | |  | |  |
| [31] |  |  | |  | |  | |  | |  | |  | |  | |  | |  | |  | |  | |  | |  | |  | |  | |  | |  | |  | |  | |  | |  | |  | |  |
| [32] |  |  | |  | |  | |  | |  | |  | |  | |  | |  | |  | |  | |  | |  | |  | |  | |  | |  | |  | |  | |  | |  | |  | |  |
| [33] |  |  | |  | |  | |  | |  | |  | |  | |  | |  | |  | |  | |  | |  | |  | |  | |  | |  | |  | |  | |  | |  | |  | |  |
| [34] |  |  | |  | |  | |  | |  | |  | |  | |  | |  | |  | |  | |  | |  | |  | |  | |  | |  | |  | |  | |  | |  | |  | |  |
| [35] |  |  | |  | |  | |  | |  | |  | |  | |  | |  | |  | |  | |  | |  | |  | |  | |  | |  | |  | |  | |  | |  | |  | |  |
| [36] |  |  | |  | |  | |  | |  | |  | |  | |  | |  | |  | |  | |  | |  | |  | |  | |  | |  | |  | |  | |  | |  | |  | |  |
| [37] |  |  | |  | |  | |  | |  | |  | |  | |  | |  | |  | |  | |  | |  | |  | |  | |  | |  | |  | |  | |  | |  | |  | |  |
| [38] |  |  | |  | |  | |  | |  | |  | |  | |  | |  | |  | |  | |  | |  | |  | |  | |  | |  | |  | |  | |  | |  | |  | |  |
| [39] |  |  | |  | |  | |  | |  | |  | |  | |  | |  | |  | |  | |  | |  | |  | |  | |  | |  | |  | |  | |  | |  | |  | |  |
| [40] |  |  | |  | |  | |  | |  | |  | |  | |  | |  | |  | |  | |  | |  | |  | |  | |  | |  | |  | |  | |  | |  | |  | |  |
|  |  |  | |  | |  | |  | |  | |  | |  | |  | |  | |  | |  | |  | |  | |  | |  | |  | |  | |  | |  | |  | |  | |  | |  |
|  |  | | No | |  | | Somewhat | |  | | Yes | |  | | N/A | |  | |  | |  | |  | |  | |  | |  | |  | |  | |  | |  | |  | |  | |  | |  | |

**Table S4** Quality assessment score of included studies using CHEQUE.

| **Study** | **Methodological quality final score** | **Reporting quality final score** |
| --- | --- | --- |
| Mervin et al. (2018)[1] | 86.5 | 83 |
| Tseng et al. (2021)[2] | 89 | 89.5 |
| Leeuwen et al. (2021)[3] | 92.5 | 96 |
| Adams et al. (2021)[4] | 84 | 72.5 |
| Areia et al. (2022)[5] | 94.5 | 88 |
| Lin et al. (2023)[6] | 80 | 95 |
| Pham et al. (2023)[7] | 87.5 | 78.5 |
| Hassan et al. (2023)[8] | 95.5 | 98 |
| Liu et al. (2023)[9] | 91.5 | 96.5 |
| Libanio et al. (2024)[10] | 94 | 93.5 |
| Wu et al. (2024)[11] | 94 | 94 |
| Hill et al. (2024)[12] | 73.5 | 83.5 |
| Curl et al. (2024)[13] | 96 | 87.5 |
| Thao et al. (2024)[14] | 80 | 92 |
| Zanghelini et al. (2024)[15] | 71.5 | 85 |
| Sun et al. (2024)[16] | 74 | 71 |
| Liu et al. (2024)[17] | 85.5 | 87 |
| Chen et al. (2025)[18] | 69 | 64.5 |
| Havreng-Théry et al. (2025)[19] | 85.5 | 75.5 |
| Maas et al. (2025)[20] | 79.5 | 82 |
| Bustamante-Balén (2025)[21] | 89.5 | 95 |
| Trujillo et al. (2025)[22] | 90 | 87 |
| Tsiachristas et al. (2025)[23] | 82 | 88 |
| Reginster et al. (2025)[24] | 93 | 88 |
| Hiligsmann et al. (2025)[25] | 92.5 | 90 |
| Du et al. (2025)[26] | 83 | 85 |
| Gandjour et al. (2025)[27] | 81 | 88 |
| Tabuchi et al. (2025)[28] | 91 | 79 |
| Boverhof et al. (2025)[29] | 89.5 | 86 |
| Huang et al. (2025)[30] | 93.5 | 90.5 |
| Kubor et al. (2025)[31] | 76.5 | 69.5 |
| Kim et al. (2025)[32] | 82.5 | 84.5 |
| Wong et al. (2025)[33] | 74.5 | 75.5 |
| Liu et al. (2025)[34] | 89 | 86 |
| Goh et al. (2026)[35] | 93 | 91 |
| Godfrey et al. (2026)[36] | 87 | 89 |
| Kim et al. (2026)[37] | 91.5 | 88.5 |
| Courtney et al. (2026)[38] | 78 | 79 |
| Hill et al. (2026)[39] | 82.5 | 78 |
| Hiligsmann et al. (2026)[40] | 83 | 87 |

**Table S5** Characteristics of included studies (n=40), first part.

| **Study** | **Population** | **Age range** | **Context** | **Targeted conditions** | **Intervention name** | **Application of AI** | **ICOPE stage** |
| --- | --- | --- | --- | --- | --- | --- | --- |
| Mervin et al. (2018)[1] | Patients with dementia | Mean age 84-86 | Australia | Dementia | PARO | Emotional support | Stage 4 (Management) |
| Tseng et al. (2021)[2] | General public | Start from 65 | United States | ALVD | Screening using AI electrocardiogram | Detection and perception | Stage 1  (Screening) |
| Leeuwen et al. (2021)[3] | Patients admitted for acute stroke | Start from 66 | United Kingdom | Stroke | AI tools for large vessel occlusion detection in emergency care | Detection and perception | Stage 2  (Diagnosis) |
| Adams et al. (2021)[4] | General public | Mean age 62 | United States | Lung cancer | AI combined with Lung-RADS | Assessment and prediction | Stage 1  (Screening) |
| Areia et al. (2022)[5] | General public | Start from 50 | United States | Colorectal cancer | Colonoscopy with AI | Detection and perception | Stage 1  (Screening) |
| Lin et al. (2023)[6] | General public | Start from 65 | China | DR | Telemedicine screening with AI | Detection and perception | Stage 1  (Screening) |
| Pham et al. (2023)[7] | Hospitalized patients | Mean age 85 | Australia | Injurious falls | The Ambient Intelligent Geriatric Management system | Assessment and prediction | Stage 4 (Management) |
| Hassan et al. (2023)[8] | Patients positive on fecal immunochemical test | Start from 50 | Italy | Colorectal cancer | AI-assisted colonoscopy | Detection and perception | Stage 2  (Diagnosis) |
| Liu et al. (2023)[9] | General public | Start from 50 | China | Blindness-causing eye diseases | Telemedicine screening with AI | Detection and perception | Stage 1  (Screening) |
| Libanio et al. (2024)[10] | General public | Start from 50 | Netherlands, Italy and Portugal | Gastric cancer | AI-assisted EGD | Detection and perception | Stage 1  (Screening) |
| Wu et al. (2024)[11] | General public | Start from 50 | China | Cataracts | AI-based digital technology-driven hierarchical screening | Detection and perception | Stage 1  (Screening) |
| Hill et al. (2024)[12] | General public | Start from 50 | United Kingdom | Breast cancer | AI-based risk-stratified screening | Assessment and prediction | Stage 1  (Screening) |
| Curl et al. (2024)[13] | General public, female | Start from 71 | United States | OVCFs | AI-based opportunistic screening strategy for OVCFs on radiographs | Detection and perception | Stage 1  (Screening) |
| Thao et al. (2024)[14] | General public | Mean age 63 | United States | Low ejection fraction | AI-guided electrocardiogram screening | Assessment and prediction | Stage 1  (Screening) |
| Zanghelini et al. (2024)[15] | Patients undergoing THA and TKA | Mean age 67.4 | United Kingdom | Osteoarthritis | GaitSmart system | Assessment and prediction | Stage 3  (Personalised treatment plan)  Stage 4 (Management) |
| Sun et al. (2024)[16] | Hospitalized patients | Mean age 59.8 | China | Malnutrition | AI-based rapid nutritional diagnostic system | Detection and perception | Stage 2  (Diagnosis) |
| Liu et al. (2024)[17] | General public | Start from 65 | Taiwan, China | ALVD | screening for ALVD using AI-enabled ECG | Detection and perception | Stage 1  (Screening) |
| Chen et al. (2025)[18] | General public | Mean age 60.8 | China | DR | Screening with AI-powered portable fundus cameras | Detection and perception | Stage 1  (Screening) |
| Havreng-Théry et al. (2025)[19] | General population aged ≥75 years | Mean age 83.8 | France | Emergency hospitalizations | Health monitoring using an eHealth system with AI | Assessment and prediction | Stage 4 (Management) |
| Maas et al. (2025)[20] | Patients diagnosed with cirrhosis | Start from 50 | Italy | Hepatocellular carcinoma | MRI with AI support | Detection and perception | Stage 2  (Diagnosis) |
| Bustamante-Balén (2025)[21] | General public | Mean age 61.32 | Spain | Colorectal cancer | An intelligent endoscopy module for computer-assisted detection and characterization | Detection and perception | Stage 2  (Diagnosis) |
| Trujillo et al. (2025)[22] | Former and current smokers | Start from 55 | Spain | Non-small cell lung cancer | Screening with LungFlag (AI) only for high-risk population | Detection and perception | Stage 1  (Screening) |
| Tsiachristas et al. (2025)[23] | Patients undergoing routine CCTA | Mean age 75 (cohort 1); mean age 59 (cohort 2) | United Kingdom | Cardiac diseases | AI-Risk Classification in routine interpretation of CCTA | Assessment and prediction | Stage 2  (Diagnosis) |
| Reginster et al. (2025)[24] | General public | Start from 50 | Germany | Osteoporosis | AI-driven chest radiographs for opportunistic osteoporosis screening | Detection and perception | Stage 1  (Screening) |
| Hiligsmann et al. (2025)[25] | Women from the general population | Start from 50 | United States | Osteoporosis | Opportunistic osteoporosis screening using chest radiographs with deep learning | Detection and perception | Stage 1  (Screening) |
| Du et al. (2025)[26] | General public | Start from 50 | Sweden | Prostate cancer | AI-assisted pathology screening | Detection and perception | Stage 1  (Screening) |
| Gandjour et al. (2025) [27] | In-center hemodialysis patients | Start from 67 | Germany | Anemia | Anemia Control Model | Decision support | Stage 3  (Personalised treatment plan) |
| Tabuchi et al. (2025)[28] | Patients undergoing ophthalmic surgeries | Mean age 69.2 (pre-intervention); mean age 67.6 (post-intervention) | Japan | Surgical errors in ophthalmology | AI-based surgery safety system | Detection and perception | Stage 4  (Treatment) |
| Boverhof et al. (2025)[29] | General public | Start from 50 | Netherlands | Glaucoma | Population-wide AI-based glaucoma screening | Detection and perception | Stage 1  (Screening) |
| Huang et al. (2025)[30] | Socioeconomically marginalized population | Mean age 59.2 | China | Lung cancer | A mobile LDCT program with an AI-based diagnostic system | Detection and perception | Stage 1  (Screening) |
| Kubor et al. (2025)[31] | End-stage cancer patients | Mean age 60.1 | Iran | Cancer | AI-driven home-based palliative care model | Operational optimization | Stage 4 (Management) |
| Kim et al. (2025)[32] | General public | Age range 54-74 | South Korea | Lung cancer | AI-assisted CXR and AI-assisted LDCT | Detection and perception | Stage 1  (Screening) |
| Wong et al. (2025)[33] | General public | Start from 50 | China | Colorectal  Cancer | AI-assisted colonoscopy | Detection and perception | Stage 1  (Screening) |
| Liu et al. (2025)[34] | Rural residents | Start from 65 | Taiwan, China | Atrial fibrillation | Screening using AI electrocardiogram | Detection and perception | Stage 1  (Screening) |
| Goh et al. (2026)[35] | Women from the general population | Start from 50 | Singapore | Breast cancer | AI-Assisted Breast Cancer Screening | Detection and perception | Stage 1  (Screening) |
| Godfrey et al. (2026)[36] | Clinical population | Start from 60 | United States | Lung cancer | AI-supported clinician risk stratification | Assessment and prediction | Stage 2  (Diagnosis) |
| Kim et al. (2026)[37] | Patients with suspected KOA | Start from 50 | South Korea | Knee osteoarthritis | AI-assisted Kellgren-Lawrence grading | Detection and perception | Stage 2  (Diagnosis) |
| Courtney et al. (2026)[38] | Patients at risk of prostate cancer | Start from 71 | United States | Prostate cancer | ArteraAI Prostate Test | Assessment and prediction | Stage 3  (Personalised treatment plan) |
| Hill et al. (2026)[39] | Women from the general population | Start from 50 | United Kingdom | Breast cancer | Integrating AI for cancer detection | Detection and perception | Stage 1  (Screening) |
| Hiligsmann et al. (2026)[40] | General public | Start from 50 | South Korea | Osteoporosis | AI-enhanced osteoporosis screening | Detection and perception | Stage 1  (Screening) |
| AI: artificial intelligence, Lung-RADS: lung CT screening reporting and data system, DR: diabetic retinopathy, EGD: esophagogastroduodenoscopy, OVCFs: osteoporotic vertebral compression fractures, THA: total hip arthroplasty, TKA: total knee arthroplasty, ALVD: asymptomatic left ventricular dysfunction, ECG: electrocardiography, MRI: magnetic resonance imaging, CCTA: coronary computed tomography angiography, LDCT: Low-dose computed tomography; CXR: chest X-ray; KOA: knee osteoarthritis. | | | | | | | |

**Table S6** Characteristics of included studies (n=40), second part.

| **Study** | **Type of analysis** | **Study design** | **Perspective** | **Comparator** | **Outcome** | **Output** | **AI-related cost** | **Source of AI cost** | **Conclusion** |
| --- | --- | --- | --- | --- | --- | --- | --- | --- | --- |
| Mervin et al. (2018)[1] | CEA | Trial-based evaluation using descriptive comparison | Healthcare payer | (1) Usual care;  (2) PARO without AI | Change in agitation level scores | ICER | USD 11.4 | Trial data | Not cost-effective |
| Tseng et al. (2021)[2] | CUA | Model-based evaluation using Decision tree and Markov model | Health sector | No screening | QALYs | ICUR | USD 59.7 | Assumption | Cost-effective |
| Leeuwen et al. (2021)[3] | CUA | Model-based evaluation using Markov model | Societal | Standard care | QALYs | ICUR | USD 46.4 | Literature | Cost-saving |
| Adams et al. (2021)[4] | Cost analysis | Model-based evaluation using Decision tree | Healthcare payer | Lung-RADS without AI | N/A | Cost savings | USD 0 | Not provided | Cost-saving |
| Areia et al. (2022)[5] | CEA | Model-based evaluation using Markov model | Societal | Screening without AI | Cancer incidence and mortality | Incremental cost and incremental health outcomes | USD 21.4 | Manufacturer | Cost-saving |
| Lin et al. (2023)[6] | CEA and CUA | Model-based evaluation using Markov model | Societal | Manual grading-based telemedicine screening for DR | Years without blindness, QALYs | ICER, ICUR | Not provided | Institutional database | Not cost-effective |
| Pham et al. (2023)[7] | CEA | Trial-based evaluation using generalized linear regression model | Health sector | Usual care | Number of injurious falls | Incremental cost and incremental health outcomes | Not provided | Trial data | Cost-saving |
| Hassan et al. (2023)[8] | CUA | Model-based evaluation using Markov model | National health system | Colonoscopy without AI | QALYs | ICUR | USD 11.4 | Assumption | Cost-saving |
| Liu et al. (2023)[9] | CEA and CUA | Model-based evaluation using Markov model | Societal | (1) No screening;  (2)Non-tele screening;  (3) tele screening without AI | Years of blindness, QALYs | ICER, ICUR | USD 7.7 | Trial data | Cost-saving |
| Libanio et al. (2024)[10] | CUA | Model-based evaluation using Markov model | Societal | (1) No screening;  (2) Screening without AI | QALYs | ICUR | USD 20.6 | Assumption | Cost-effective |
| Wu et al. (2024)[11] | CEA and CUA | Model-based evaluation using Markov model | Societal | (1) No screening;  (2) Tele screening without AI | Years of blindness, QALYs | ICER, ICUR | USD 8.9 | Trial data | Cost-saving |
| Hill et al. (2024)[12] | CUA | Model-based evaluation using discrete event simulation | Healthcare payer | Screening without AI | QALYs | INMB | USD 0 | Not provided | Cost-saving |
| Curl et al. (2024)[13] | CUA | Model-based evaluation using Decision tree and Markov model | Societal | No screening | QALYs | ICUR | USD 10.6 | Assumption, expert consultation | Cost-saving |
| Thao et al. (2024)[14] | CUA | Model-based evaluation using Decision tree and Markov model | Healthcare payer | No screening | QALYs | ICUR | USD 112.6 | Assumption | Cost-effective |
| Zanghelini et al. (2024)[15] | CUA | Model-based evaluation using Decision tree model and trial data | Healthcare payer | Standard care | QALYs | ICUR | USD 18.3 | Manufacturer | Cost-saving |
| Sun et al. (2024)[16] | CEA | Model-based evaluation using Decision tree model and trial data | Societal | Standard care | Cure rate | ICER | USD 1.2 | Trial data | Cost-effective |
| Liu et al. (2024)[17] | CUA | Model-based evaluation using Decision tree and Markov model | Healthcare payer | No screening | QALYs | ICUR | USD 5.3 | Assumption | Cost-saving |
| Chen et al. (2025)[18] | CUA | Model-based evaluation using Markov model and trial data | Societal | Screening without AI | QALYs | ICUR | USD 1.9 | Trial data | Cost-saving |
| Havreng-Théry et al. (2025)[19] | CEA | Trial-based evaluation using descriptive comparison | Healthcare payer | Usual care | Cumulative incidence of emergency hospitalizations | Incremental cost and incremental health outcomes | USD 0 | Not provided | Cost-saving |
| Maas et al. (2025)[20] | CUA | Model-based evaluation using Decision tree and Markov model | Health sector | MRI without AI | QALYs | ICUR | USD 333.7 | Assumption and expert consultation | Cost-effective |
| Bustamante-Balén (2025)[21] | CUA | Model-based evaluation using Markov model | Healthcare payer | Standard care | QALYs | ICUR | USD 8.2 | Assumption | Cost-saving |
| Trujillo et al. (2025)[22] | CUA | Model-based evaluation using Decision tree and Markov model | National health system | (1) No screening;  (2) Screening without AI | QALYs | ICUR | USD 0.02 | Literature | Cost-saving |
| Tsiachristas et al. (2025)[23] | CUA | Model-based evaluation using Decision tree and Markov model | Healthcare payer | Standard care | QALYs | ICUR | USD 318.3 | Assumption | Cost-effective |
| Reginster et al. (2025)[24] | CUA | Model-based evaluation using Decision tree and Markov model | Healthcare payer | No screening | QALYs | ICUR | USD 10.4 | Assumption | Cost-effective |
| Hiligsmann et al. (2025)[25] | CUA | Model-based evaluation using Decision tree and Markov model | Societal | No screening | QALYs | ICUR | USD 19.9 | Assumption | Cost-effective |
| Du et al. (2025)[26] | CUA | Model-based evaluation using Decision tree and Markov model | Health sector | Screening without AI | QALYs | ICUR | USD 11.5 | Assumption, literature, expert consultation | Cost-effective |
| Gandjour et al. (2025) [27] | CUA | Model-based evaluation using Markov model | Healthcare payer | Standard care | QALYs | ICUR, INMB | USD 0 | Assumption | Cost-saving |
| Tabuchi et al. (2025)[28] | CEA and CBA | Trial-based evaluation using economic simulation model | Healthcare payer | Usual care | Number of medical errors prevented | ICER | USD 24.6 | Trial data, literature, and assumption | Cost-effective |
| Boverhof et al. (2025)[29] | CUA | Model-based evaluation using Decision tree and Markov model | Societal | Opportunistic screening without AI | QALYs | ICUR | Not provided | Expert consultation | Cost-effective |
| Huang et al. (2025)[30] | CEA | Trial-based evaluation using descriptive comparison | Societal | No screening | Cancer detection rate and diagnosis rate | CER, EDCI | Not provided | Not provided | Cost-effective |
| Kubor et al. (2025)[31] | CEA | Trial-based evaluation using descriptive comparison | National health system | Usual care | Length of stay, the proportion of deaths occurring at home | ICER | Not provided | Not provided | Cost-saving |
| Kim et al. (2025)[32] | CUA | Model-based evaluation using Decision tree and Markov model | National health system | (1) No screening;  (2) screening without AI | QALYs | ICUR | USD 0.9 | Internal data | Cost-effective |
| Wong et al. (2025)[33] | CEA | Model-based evaluation using Decision tree and Markov model | Societal | No screening | Life-year saved | ICER | USD 0 | Assumption | Cost-effective |
| Liu et al. (2025)[34] | CUA | Model-based evaluation using Decision tree and Markov model | Healthcare payer | No screening | QALYs | ICUR | USD 5.1 | Assumption | Cost-effective |
| Goh et al. (2026)[35] | CUA | Model-based evaluation using Markov model | National health system | Screening without AI | QALYs | ICUR | USD 3.9 | Assumption and expert consultation | Cost-saving |
| Godfrey et al. (2026)[36] | CEA | Model-based evaluation using Decision tree | Healthcare payer | Assessment without AI | Life-year gained | ICER | USD 710.3 | National reimbursement schedule | Cost-effective |
| Kim et al. (2026)[37] | CUA | Model-based evaluation using Decision tree and Markov model | National health system | Assessment without AI | QALYs | ICUR | USD 6.8 | Assumption | Cost-saving |
| Courtney et al. (2026)[38] | CUA | Model-based evaluation using Markov model | Healthcare payer | Usual care | QALYs | ICUR | USD 685.4 | Manufacturer | Cost-saving |
| Hill et al. (2026)[39] | CUA | Model-based evaluation using discrete event simulation | National health system | Screening without AI | QALYs | ICUR | USD 10.8 | Trial data | Cost-saving |
| Hiligsmann et al. (2026)[40] | CUA | Model-based evaluation using Decision tree and Markov model | Healthcare payer | No screening | QALYs | ICUR | USD 10.2 | Assumption | Cost-effective |
| (1) AI: artificial intelligence, CEA: cost-effectiveness analysis, CUA: cost-utility analysis, CBA: cost-benefit analysis, QALY: quality-adjusted life year, ICER: incremental cost-effectiveness ratio, ICUR: incremental cost-utility ratio, INMB: incremental net monetary benefit, CER: Cost-effective Ratio, EDCI: Early Detection Cost Index, the ratio of CER to the per capita GDP; (2) Costs are presented in 2024 USD. | | | | | | | | | |

**Figure S1** Distribution of included studies focusing on patients aged 65 and above, by targeted health condition and cost-effectiveness, mapped across the ICOPE care pathway. Citation numbers correspond to the numbering used in the main text.

**REFERENCES**

1.	Mervin MC, Moyle W, Jones C, Murfield J, Draper B, Beattie E, et al. The Cost-Effectiveness of Using PARO, a Therapeutic Robotic Seal, to Reduce Agitation and Medication Use in Dementia: Findings from a Cluster–Randomized Controlled Trial. Journal of the American Medical Directors Association. 2018 Jul 1;Focus on Care of Persons with Alzheimer’s Disease and Related Dementias19(7):619-622.e1. doi:10.1016/j.jamda.2017.10.008

2.	Tseng AS, Thao V, Borah BJ, Attia IZ, Medina Inojosa J, Kapa S, et al. Cost Effectiveness of an Electrocardiographic Deep Learning Algorithm to Detect Asymptomatic Left Ventricular Dysfunction. Mayo Clin Proc. 2021 Jul;96(7):1835–44. doi:10.1016/j.mayocp.2020.11.032 PubMed PMID: 34116837.

3.	van Leeuwen KG, Meijer FJA, Schalekamp S, Rutten MJCM, van Dijk EJ, van Ginneken B, et al. Cost-effectiveness of artificial intelligence aided vessel occlusion detection in acute stroke: an early health technology assessment. Insights Imaging. 2021 Sep 25;12(1):133. doi:10.1186/s13244-021-01077-4

4.	Adams SJ, Mondal P, Penz E, Tyan CC, Lim H, Babyn P. Development and Cost Analysis of a Lung Nodule Management Strategy Combining Artificial Intelligence and Lung-RADS for Baseline Lung Cancer Screening. J Am Coll Radiol. 2021 May;18(5):741–51. doi:10.1016/j.jacr.2020.11.014 PubMed PMID: 33482120.

5.	Areia M, Mori Y, Correale L, Repici A, Bretthauer M, Sharma P, et al. Cost-effectiveness of artificial intelligence for screening colonoscopy: a modelling study. The Lancet Digital Health. 2022 Jun 1;4(6):e436–44. doi:10.1016/S2589-7500(22)00042-5 PubMed PMID: 35430151.

6.	Lin S, Ma Y, Xu Y, Lu L, He J, Zhu J, et al. Artificial Intelligence in Community-Based Diabetic Retinopathy Telemedicine Screening in Urban China: Cost-effectiveness and Cost-Utility Analyses With Real-world Data. JMIR Public Health Surveill. 2023 Feb 23;9:e41624. doi:10.2196/41624 PubMed PMID: 36821353; PubMed Central PMCID: PMC9999255.

7.	Pham CT, Visvanathan R, Strong M, Wilson ECF, Lange K, Dollard J, et al. Cost-Effectiveness and Value of Information Analysis of an Ambient Intelligent Geriatric Management (AmbIGeM) System Compared to Usual Care to Prevent Falls in Older People in Hospitals. Appl Health Econ Health Policy. 2023 Mar;21(2):315–25. doi:10.1007/s40258-022-00773-6 PubMed PMID: 36494574.

8.	Hassan C, Povero M, Pradelli L, Spadaccini M, Repici A. Cost-utility analysis of real-time artificial intelligence-assisted colonoscopy in Italy. Endosc Int Open. 2023 Nov;11(11):E1046–55. doi:10.1055/a-2136-3428 PubMed PMID: 37954109; PubMed Central PMCID: PMC10637858.

9.	Liu H, Li R, Zhang Y, Zhang K, Yusufu M, Liu Y, et al. Economic evaluation of combined population-based screening for multiple blindness-causing eye diseases in China: a cost-effectiveness analysis. The Lancet Global Health. 2023 Mar 1;11(3):e456–65. doi:10.1016/S2214-109X(22)00554-X PubMed PMID: 36702141.

10.	Libanio D, Antonelli G, Marijnissen F, Spaander MC, Hassan C, Dinis-Ribeiro M, et al. Combined gastric and colorectal cancer endoscopic screening may be cost-effective in Europe with the implementation of artificial intelligence: an economic evaluation. Eur J Gastroenterol Hepatol. 2024 Feb 1;36(2):155–61. doi:10.1097/MEG.0000000000002680 PubMed PMID: 38131423.

11.	Wu X, Wu Y, Tu Z, Cao Z, Xu M, Xiang Y, et al. Cost-effectiveness and cost-utility of a digital technology-driven hierarchical healthcare screening pattern in China. Nat Commun. 2024 Apr 30;15(1):3650. doi:10.1038/s41467-024-47211-w

12.	Hill H, Roadevin C, Duffy S, Mandrik O, Brentnall A. Cost-Effectiveness of AI for Risk-Stratified Breast Cancer Screening. JAMA Netw Open. 2024 Sep 5;7(9):e2431715. doi:10.1001/jamanetworkopen.2024.31715 PubMed PMID: 39235813; PubMed Central PMCID: PMC11377997.

13.	Curl PK, Jacob A, Bresnahan B, Cross NM, Jarvik JG. Cost-Effectiveness of Artificial Intelligence-Based Opportunistic Compression Fracture Screening of Existing Radiographs. J Am Coll Radiol. 2024 Sep;21(9):1489–96. doi:10.1016/j.jacr.2023.11.029 PubMed PMID: 38527641; PubMed Central PMCID: PMC11381181.

14.	Thao V, Zhu Y, Tseng AS, Inselman JW, Borah BJ, McCoy RG, et al. Cost-Effectiveness of Artificial Intelligence-Enabled Electrocardiograms for Early Detection of Low Ejection Fraction: A Secondary Analysis of the Electrocardiogram Artificial Intelligence-Guided Screening for Low Ejection Fraction Trial. Mayo Clin Proc Digit Health. 2024 Dec;2(4):620–31. doi:10.1016/j.mcpdig.2024.10.001 PubMed PMID: 40206537; PubMed Central PMCID: PMC11975989.

15.	Zanghelini F, Ponzo A, Xydopoulos G, Fordham R, Khanal S. Cost-Effectiveness of GaitSmart and an Artificial Intelligence Solution for Rehabilitation of Patients Undergoing Total Hip Arthroplasty (THA) and Total Knee Arthroplasty (TKA) in Older Population in the United Kingdom. Geriatrics (Basel). 2024 Oct 5;9(5):129. doi:10.3390/geriatrics9050129 PubMed PMID: 39451861; PubMed Central PMCID: PMC11508099.

16.	Sun MY, Wang Y, Zheng T, Wang X, Lin F, Zheng LY, et al. Health economic evaluation of an artificial intelligence (AI)-based rapid nutritional diagnostic system for hospitalised patients: A multicentre, randomised controlled trial. Clinical Nutrition. 2024 Oct 1;43(10):2327–35. doi:10.1016/j.clnu.2024.08.030

17.	Liu WT, Hsieh PH, Lin CS, Fang WH, Wang CH, Tsai CS, et al. Opportunistic Screening for Asymptomatic Left Ventricular Dysfunction With the Use of Electrocardiographic Artificial Intelligence: A Cost-Effectiveness Approach. Can J Cardiol. 2024 Jul;40(7):1310–21. doi:10.1016/j.cjca.2023.11.044 PubMed PMID: 38092190.

18.	Chen Y, Song F, Zhao Z, Wang Y, To E, Liu Y, et al. Acceptability, applicability, and cost-utility of artificial-intelligence-powered low-cost portable fundus camera for diabetic retinopathy screening in primary health care settings. Diabetes Res Clin Pract. 2025 May;223:112161. doi:10.1016/j.diabres.2025.112161 PubMed PMID: 40194705.

19.	Havreng-Théry C, Fouchard A, Denis F, Veyron JH, Belmin J. Cost-Effectiveness Analysis of a Machine Learning-Based eHealth System to Predict and Reduce Emergency Department Visits and Unscheduled Hospitalizations of Older People Living at Home: Retrospective Study. JMIR Form Res. 2025 Apr 11;9:e63700. doi:10.2196/63700 PubMed PMID: 40215100; PubMed Central PMCID: PMC12032495.

20.	Maas L, Contreras-Meca C, Ghezzo S, Belmans F, Corsi A, Cant J, et al. Cost-effectiveness analysis of artificial intelligence (AI) in earlier detection of liver lesions in cirrhotic patients at risk of hepatocellular carcinoma in Italy. Journal of Medical Economics. 2025 Jul 11;28(1):1023–36. doi:10.1080/13696998.2025.2525006 PubMed PMID: 40586549.

21.	Bustamante-Balén M, Merino Rodríguez B, Barranco L, Monje J, Álvarez M, de Pedro S, et al. Cost-effectiveness analysis of artificial intelligence-aided colonoscopy for adenoma detection and characterization in Spain. Endosc Int Open. 2025 Mar 14;13:a25097278. doi:10.1055/a-2509-7278 PubMed PMID: 40109314; PubMed Central PMCID: PMC11922311.

22.	Trujillo JC, Soriano JB, Marzo M, Higuera O, Gorospe L, Pajares V, et al. Cost-effectiveness of a machine learning risk prediction model (LungFlag) in the selection of high-risk individuals for non-small cell lung cancer screening in Spain. J Med Econ. 2025 Dec;28(1):147–56. doi:10.1080/13696998.2024.2444781 PubMed PMID: 39697091.

23.	Tsiachristas A, Chan K, Wahome E, Kearns B, Patel P, Lyasheva M, et al. Cost-effectiveness of a novel AI technology to quantify coronary inflammation and cardiovascular risk in patients undergoing routine coronary computed tomography angiography. Eur Heart J Qual Care Clin Outcomes. 2025 Jun 1;11(4):434–44. doi:10.1093/ehjqcco/qcae085

24.	Reginster JY, Schmidmaier R, Alokail M, Hiligsmann M. Cost-effectiveness of opportunistic osteoporosis screening using chest radiographs with deep learning in Germany. Aging Clin Exp Res. 2025 May 13;37(1):149. doi:10.1007/s40520-025-03048-x PubMed PMID: 40355760; PubMed Central PMCID: PMC12069426.

25.	Hiligsmann M, Silverman SL, Reginster JY. Cost-Effectiveness of Opportunistic Osteoporosis Screening Using Chest Radiographs With Deep Learning in the United States. J Am Coll Radiol. 2025 Nov;22(11):1346–56. doi:10.1016/j.jacr.2025.07.028 PubMed PMID: 40780311.

26.	Du X, Hao S, Olsson H, Kartasalo K, Mulliqi N, Rai B, et al. Effectiveness and Cost-effectiveness of Artificial Intelligence-assisted Pathology for Prostate Cancer Diagnosis in Sweden: A Microsimulation Study. Eur Urol Oncol. 2025 Feb;8(1):80–6. doi:10.1016/j.euo.2024.05.004 PubMed PMID: 38789385.

27.	Gandjour A, Apel C, Kendzia D, Neri L, Bellocchio F, Usvyat L, et al. Health-economic evaluation of an AI-powered decision support system for anemia management in in-center hemodialysis patients. BMC Nephrol. 2025 Aug 28;26(1):496. doi:10.1186/s12882-025-04298-7

28.	Tabuchi H, Ishitobi N, Deguchi H, Nakaniida Y, Tanaka H, Akada M, et al. Large-scale observational study of AI-based patient and surgical material verification system in ophthalmology: real-world evaluation in 37 529 cases. BMJ Qual Saf. 2025 Jun 19;34(7):433–42. doi:10.1136/bmjqs-2024-018018 PubMed PMID: 39613452; PubMed Central PMCID: PMC12229072.

29.	Boverhof BJ, Ramos IC, Vermeer KA, Vries VA de, Klaver CCW, Ramdas WD, et al. The Cost-Effectiveness of an Artificial Intelligence-Based Population-Wide Screening Program for Primary Open-Angle Glaucoma in The Netherlands. Value in Health. 2025 Sep 1;28(9):1317–26. doi:10.1016/j.jval.2025.06.006 PubMed PMID: 40581156.

30.	Huang F, Lin X, Hong Y, Li Y, Li Y, Chen WT, et al. The feasibility and cost-effectiveness of implementing mobile low-dose computed tomography with an AI-based diagnostic system in underserved populations. BMC Cancer. 2025 Feb 25;25(1):345. doi:10.1186/s12885-025-13710-2 PubMed PMID: 40001094; PubMed Central PMCID: PMC11863806.

31.	Kubor P, Pourghaderi AR, Moeeni M, Rezayatmand R. Cost-effectiveness analysis of home-based palliative care for end-stage cancer patients. Cost Eff Resour Alloc. 2025 Dec 25;24(1):15. doi:10.1186/s12962-025-00699-5

32.	Kim K, Kim JH, Shin J, Kim MS, Park SH, Chang JH, et al. Cost-effectiveness of chest radiography using artificial intelligence for lung cancer screening in South Korea. Sci Rep. 2025 Nov 28;15(1):45604. doi:10.1038/s41598-025-29600-3

33.	Wong MC, Huang J, Lam TY, Lau LH, Chiu PW. The Cost-Effectiveness of AI-Assisted Colonoscopy as a Primary or Secondary Screening Test in a Population-Based Colorectal Cancer Screening Program: Markov Modeling–Based Cost Effectiveness Analysis. J Med Internet Res. 2025 Sep 26;27:e67762. doi:10.2196/67762

34.	Liu WT, Lin CS, Lin C, Lin TK, Lin WY, Lee CC, et al. Universal Atrial Fibrillation Screening Using Electrocardiographic Artificial Intelligence: A Cost-Effective Approach in Rural Communities. J Med Syst. 2025 Oct 27;49(1):145. doi:10.1007/s10916-025-02287-9

35.	Goh SSN, Lim YZ, Ong C, Hartman M, Wang Y. Cost Effectiveness Analysis of an AI-Assisted Breast Cancer Screening Programme in Singapore: An Early Health Technology Assessment. Cancers. 2026 Mar 4;18(5):836. doi:10.3390/cancers18050836

36.	Godfrey CM, Leech AA, McGann KC, Zhu J, Marmor HN, Pena S, et al. Cost-effectiveness analysis of artificial intelligence-assisted risk stratification of indeterminate pulmonary nodules. Lim JH, editor. PLoS One. 2026 Mar 5;21(3):e0343492. doi:10.1371/journal.pone.0343492

37.	Kim K, Kim SY, Kim SS, Kim JH, Ro DH, Choi BS. Cost-Effectiveness of AI-Assisted Kellgren-Lawrence Grading of Knee Osteoarthritis in the South Korean Health-Care System. Journal of Bone and Joint Surgery. 2026 Jun 3. doi:10.2106/JBJS.25.01278

38.	Courtney PT, Shih YCT, Chang AJ, Lee A, Steinberg ML, Valle LF, et al. Cost-effectiveness of an artificial intelligence predictive model for guiding androgen deprivation therapy in intermediate-risk prostate cancer. JNCI Cancer Spectrum. 2026 Mar 3;10(2):pkag035. doi:10.1093/jncics/pkag035

39.	Hill H, Roadevin C. Economic evaluation of artificial intelligence for cancer detection in the UK breast screening programme. Br J Cancer. 2026 May 2. doi:10.1038/s41416-026-03465-3

40.	Hiligsmann M, Kong SH, Alokail M, Kim MY, Reginster JY. Evaluating the cost-effectiveness of artificial intelligence-enhanced osteoporosis screening in men and women using routine chest radiographs in South Korea. JBMR Plus. 2026 Jan 9;10(2):ziaf187. doi:10.1093/jbmrpl/ziaf187
